# Kinship verification and recognition based on handcrafted and deep learning feature-based techniques

**DOI:** 10.7717/peerj-cs.735

**Published:** 2021-12-06

**Authors:** Nermeen Nader, Fatma El-Zahraa El-Gamal, Shaker El-Sappagh, Kyung Sup Kwak, Mohammed Elmogy

**Affiliations:** 1 Information Technology Department, Faculty of Computers and Information, Mansoura University, Mansoura, Egypt; 2 Information Systems Department, Faculty of Computers and Artificial Intelligence, Benha University, Banha, Egypt; 3 Faculty of Computer Science and Engineering, Galala University, Suez, Egypt; 4 Department of Information and Communication Engineering, Inha University, Incheon, South Korea

**Keywords:** Kinship verification, Kinship recognition, Handcrafted feature-based techniques, Deep learning techniques, Benchmark datasets

## Abstract

**Background and Objectives:**

Kinship verification and recognition (KVR) is the machine’s ability to identify the genetic and blood relationship and its degree between humans’ facial images. The face is used because it is one of the most significant ways to recognize each other. Automatic KVR is an interesting area for investigation. It greatly affects real-world applications, such as searching for lost family members, forensics, and historical and genealogical studies. This paper presents a comprehensive survey that describes KVR applications and kinship types. It presents a literature review of current studies starting from handcrafted passing through shallow metric learning and ending with deep learning feature-based techniques. Furthermore, kinship mostly used datasets are discussed that in turn open the way for future directions for the research in this field. Also, the KVR limitations are discussed, such as insufficient illumination, noise, occlusion, and age variations problems. Finally, future research directions are presented, such as age and gender variation problems.

**Methods:**

We applied a literature survey methodology to retrieve data from academic databases. An inclusion and exclusion criteria were set. Three stages were followed to select articles. Finally, the main KVR stages, along with the main methods in each stage, were presented. We believe that surveys can help researchers easily to detect areas that require more development and investigation.

**Results:**

It was found that handcrafted, metric learning, and deep learning were widely utilized in kinship verification and recognition problem using facial images.

**Conclusions:**

Despite the scientific efforts that aim to address this hot research topic, many future research areas require investigation, such as age and gender variation. In the end, the presented survey makes it easier for researchers to identify the new areas that require more investigation and research.

## Introduction

Nowadays, analyzing facial images is considered a hot and interesting topic of study and research in pattern recognition, computer vision, and image processing. This consideration is due to the availability of much socially-relevant information in the human face, such as gender, age, and emotional state (Duan, Zhang & Zuo, 2017). Furthermore, the face conveys many discriminating features enough to determine a person’s identity (Arachchilage & Izquierdo, 2020; Wang & Deng, 2021). Among the research areas that depend on facial image analysis is computer-based kinship verification and recognition (KVR).

Kinship is the ability to understand the genetic and blood bond between parent-child, sibling-sibling, and grandparent-child members of family (Xu & Shang, 2016). A parent shares an offspring with much more than half of his/her genes (Harpending, 2002). Therefore, there are different types of kinship ties (Jha, 1994). Genetic and blood kinship, such as parents-children, are called blood ties. Marriage relationship (*i.e*., husband and wife) forms the marriage tie. Also, there are various degrees of kinship: primary, secondary, and tertiary kinships. Kin directly related to one another, such as mother-child, father-child, and sister-brother, is called primary kinship. Secondary kinship means the primary kin of the first-degree kin, such as uncles, sister’s husband, and brother’s wife. Tertiary kinship refers to the primary kin’s secondary kin like the brother of sister’s husband (Almuashi et al., 2017).

Artificial intelligence is an interesting field which takes advantage of the fact that computers are unable to distinguish between numerical and symbolic representations, allowing them to perform symbol processing as easily as numerical processing (Simmons & Chappell, 1988). It can also be defined as a digital computer’s or a computer-controlled robot’s ability to perform tasks ordinarily performed by intelligent people. Deep learning is a subcategory of machine learning, which is itself a subcategory of AI. As a result, everything classified as deep learning or machine learning falls within the AI umbrella. AI can be used in many useful applications such as Chronic Obstructive Pulmonary Disease (COPD) disease detection (Srivastava et al., 2021), stance detection (Karande et al., 2021), cervical cancer detection (Bhatt, Ganatra & Kotecha, 2021), stock price prediction (Patel et al., 2015), spam detection (Sanghani & Kotecha, 2019), Kidney disease detection (Zeynu & Patil, 2018) and Fashion Analysis (Wazarkara, Patilb & Kumarc, 2020).

**Figure 1:**
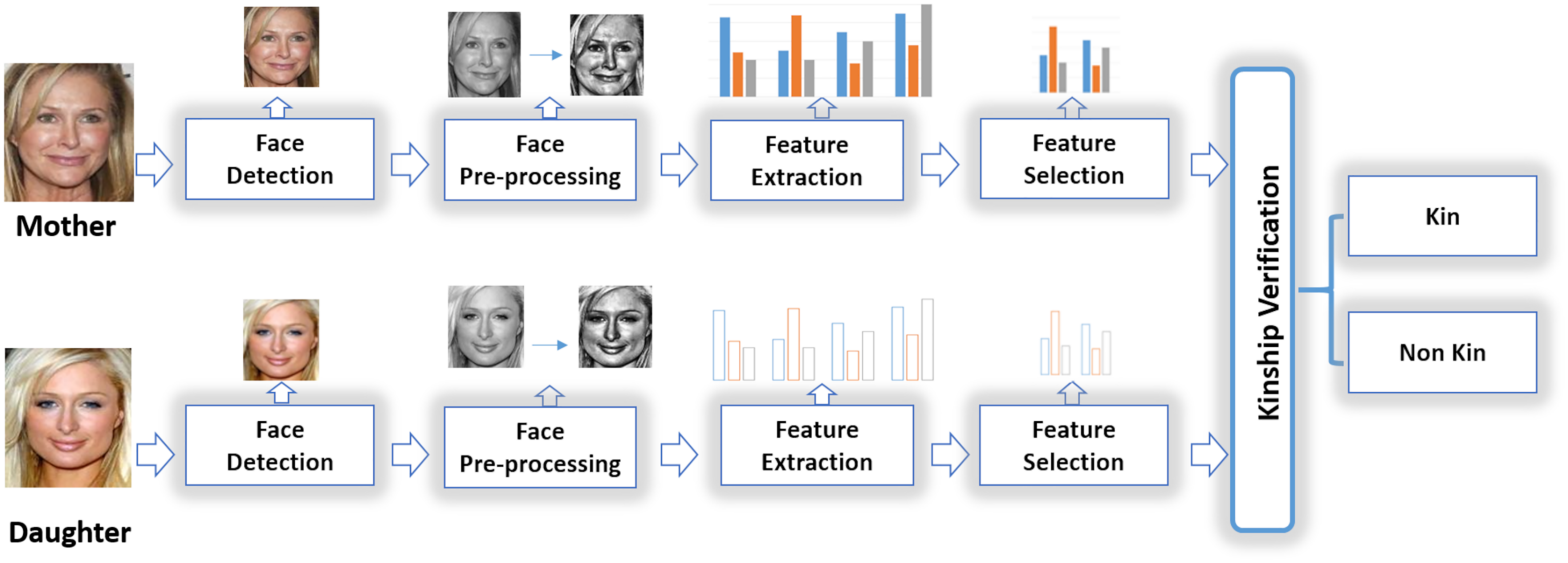
A general KVR framework (Ruogu et al., 2013).

The automatic KVR is an interesting and novel area of research within computer vision and AI. The automatic KVR systems have, in general, two goals. The first is the verification goal. The target here is to show the machine’s ability to discriminate kin from family members (*i.e*., checking the presence of genetic or blood relationship between the facial images). The second goal is the recognition that aims to identify the degree of relationship (Almuashi et al., 2017). Figure 1 shows a framework example of the KVR system, which indicates two input images (the images are provided from Family 101 dataset (Fang et al., 2013)) on which the existence of a genetic relationship and its degree need to be verified. The system consists of various stages, such as face detection, face preprocessing, feature extraction, feature selection, and kinship verification, to have a final decision whether the two images kin or not. Furthermore, if the two facial images have a kin relationship, we can identify the degree of relationship (recognition). Based on its goals, the automatic KVR systems can be used in many applications, such as family members identification from an image archive, forensics, historical/genealogical studies, and finding lost children/parents (Wu et al., 2016; Guo & Wang, 2012).

Many people get confused between kinship and face recognition systems as they depend on facial image analysis. Face recognition refers to whether two persons are the same or not by individual’s photograph comparison with album photograph (Shinwari et al., 2019; Wei & Li, 2017; Schroff, Kalenichenko & Philbin, 2015). A face recognition system can perform either facial verification or identification. Facial verification is the ability to know that the two images belong to the same person. In other words, it is the classification of two images as they belong to the same person or not.

**Figure 2:**
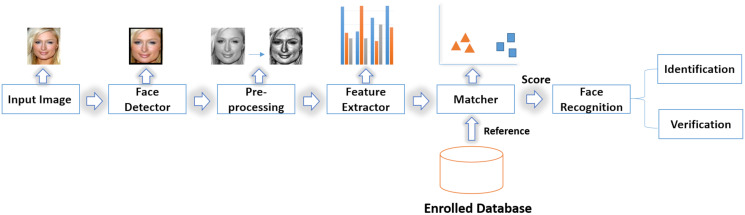
A common face recognition framework (Ruogu et al., 2013).

On the other hand, facial identification means recognizing the identity of the person (Fontaine, Achanta & Süsstrunk, 2017). Figure 2 represents face recognition system framework where the face detector detects face area within the image (the images are provided from Family 101 dataset (Fang et al., 2013)). Using the preprocessed detected face, a feature extractor is used to extract facial features and input them to the matcher for comparing it with the features of database images. The matcher produces a score of similarity between the input face and each registered face. This score can be used for the recognition process that can be in the mode of identification or verification (Wei & Li, 2017). Facial recognition systems can also be used in many applications, advertisements, healthcare, security, and criminal recognition (Shinwari et al., 2019). As the face recognition system is used as a security system that can secure our homes, offices, the face recognition system needs to be more secure. A traditional safety system has Internet of Things (IoT) flaws and is occasionally unable to deliver real-time data (Sayem & Chowdhury, 2018). Consequently, IoT security algorithms are used to secure the face recognition system as in Patil, Joshi & Patil (2020) and Patil & Joshi (2019).

**Figure 3:**
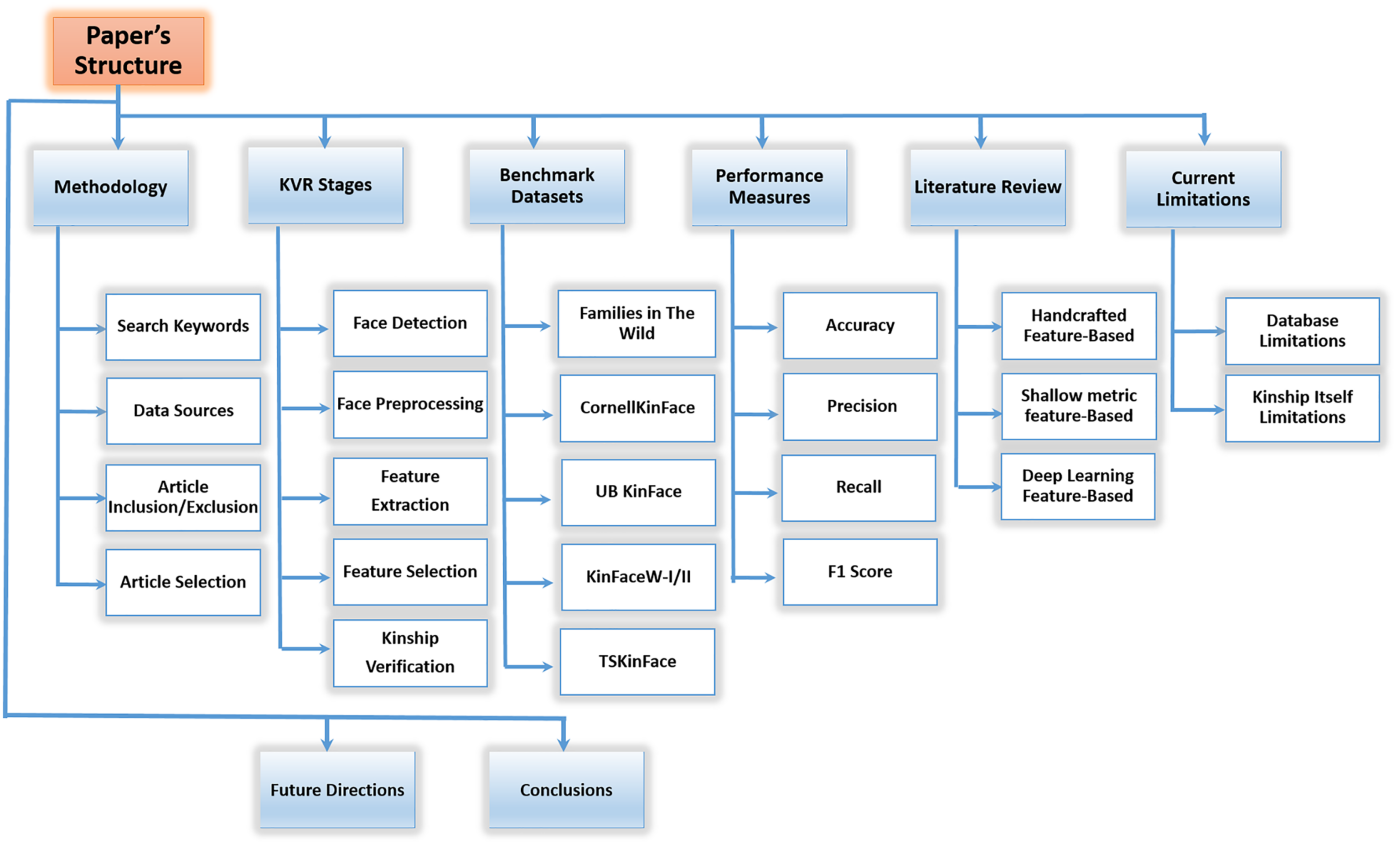
The presented survey structure.

It is a complicated task to analyze kinship through a human facial image. Mainly, the limitations in identifying kin relationships can be categorized into two classes: (1) kinship itself limitations, such as age and gender variations. (2) database environment limitations, such as pose variation, illumination variations, facial expression, and background clutter (Almuashi et al., 2017; Mun, Deorankar & Chatur, 2014; Lamba et al., 2011; Lin, 2000; Biswas, Bowyer & Flynn, 2011; Bottinok, Islam & Vieira, 2015; Tan et al., 2006). These limitations mainly affect extracting features that are so sensitive and important in kinship verification and recognition tasks. Therefore, this paper aims to show a comprehensive survey about the current researches in kinship verification analysis field. Furthermore, recommendations to some new research directions are made like deep learning and new techniques, such as GAN and reinforcement learning. For this purpose, the remainder of this article is arranged as in the following sections: “Methodology” explains the methodology for the survey, such as search keywords, data sources, and article selection. “KVR Stages” shows the complete stages of the automatic KVR system, including the most popular techniques used in each stage. “Benchmark Datasets” shows popular kinship-based benchmark datasets. “Performance Measures” presents different system performance measures, such as accuracy, and recall. “Literature Review” presents a literature review of kinship current studies starting from handcrafted features passing through shallow metric learning and ending with deep learning feature-based techniques. “Current Limitations” discusses kinship limitations, such as database limitations, and kinship itself limitations. “Future Directions” presents future directions, such as age and gender variation. “Conclusions” is the last section of the survey. Figure 3 describes the structure of these sections.

## Methodology

This section explains the protocol used to survey the different aspects used to solve the kinship verification problem from the year 2010 to the year 2021. The search keywords, data sources, inclusion/exclusion criteria, and article selection used are discussed. Tables 1 and 2 represents frequency based analysis of the techniques and sub-techniques used for kinship verification.

**Table 1:** Frequency-based analysis of technique types.

No.	Method type	Method frequency %
1	Handcafted feature-based	30
2	Shallow metric feature-based	30
3	Deep learning feature-based	40

**Table 2:** Frequency-based analysis of sub-technique types.

No.	Method type	CLBP	HOG	LBP	SIFT	TPLBP	LPQ	SVM	KNN	CNN
1	Handcrafted feature-based	8%	20%	12%	–	–	12%	36%	12%	–
2	Shallow metric feature-based	–	13%	27%	20%	13%	5%	17%	5%	–
3	Deep learning feature-based	–	–	10%	–	–	–	30%	–	60%

### Search keywords

The keywords for the initial search were carefully chosen. Following an initial search, new words found in various relevant articles were used to create numerous keywords. The most appropriate key-phrases that were mentioned in many research papers have been selected as initial keywords such as (‘Kinship verification’, ‘Kinship discriminative features’, ‘Kinship face analysis’, ‘mid-level features kinship verification’). Furthermore, other keywords were selected depending on our understanding of the topic such as (‘Low-level features kinship verification’, ‘High-level features kinship verification’, ‘Facial kinship verification’, ‘Handcrafted kinship verification’, ‘metric learning kinship verification’ and ‘Deep learning kinship verification’).

### Data sources

Table 3 lists the several academic databases that were used to find articles for the survey.

**Table 3:** Academic databases.

Academic database	Link
IEEEXplore	https://ieeexplore.ieee.org/
Sciencedirect	http://www.sciencedirect.com/
Springerlink	https://link.springer.com/
Web of science	https://apps.webofknowledge.com/
Scopus	https://www.scopus.com/

### Article inclusion/exclusion criteria

To determine which publications are suitable for the next review stage, inclusion/exclusion criteria were established based on the research goal. Articles that met the inclusion criteria were considered relevant to the study, while those that didn’t meet the requirements were eliminated. The set of inclusion/exclusion criteria are provided in Table 4.

**Table 4:** Article inclusion and exclusion criteria.

Inclusion criteria	Exclusion criteria
The review only focuses on kinship verification using facial images	Articles that use other than facial images were excluded
Kinship verification methods based on 3 categories (handcrafted, metric learning, and deep learning) were considered	Other categories of kinship verification methods such as (feature-based and model-based) are not considered
Only articles written in the English language were considered for inclusion	Articles written in languages other than English were excluded

### Article selection

In order to select an article for this research, three stages were followed. The first stage was that only the articles’ titles, abstracts and keywords were considered to extract relevant articles so that the initial number of articles considered was 53. The second stage was that the abstract, introduction, and conclusion were analyzed to refine the first stage’s choice. The number of the articles after applying this stage was reduced from 53 to 41. At the final stage, the articles were read thoroughly, and then the quality of the articles was rated depending on their relevance to the research. The final number of the selected articles is 30.

## KVR stages

As mentioned before, our main concern is automatic kinship verification. To verify the existence of the kin relationship between two persons using facial images, Table 5 shows the 5 stages that need to be processed. The following subsections describe each stage in details.

**Table 5:** KVR stages.

Stage No.	KVR stages
1.	Face detection
2.	Face preprocessing
3.	Feature extraction
4.	Feature selection
5.	Kinship verification

### Face detection

It is a principle stage of the automatic system of kinship verification. It is the detection and extraction process of facial patches from images. An effective face detection technique must be robust to light, position, orientation, and other face detection limitations. Specific techniques that can be used for face detection from an image are (Almuashi et al., 2017): active appearance model (AAM) (Ghahramani, Yau & Teoh, 2014), boosted cascade of simple (Guo & Wang, 2012), Adaboost face detector Kohli, Singh & Vatsa, 2012, Fiducial points detection (Xia, Shao & Fu, 2012), Viola and Jones algorithm (Chergui et al., 2020), skin color model-based algorithm (Sahu & Dash, 2020), Successive Mean Quantization Transform (SMQT) Features and Sparse Network of Winnows (SNOW) Classifier Method (Nilsson, Nordberg & Claesson, 2007) and Neural Network-Based Face Detection (Dang & Sharma, 2017).

### Face preprocessing

This stage aims to make the facial input image conditions similar to those stored in the dataset. The aim is to align images and minimize illumination, posture, orientation, and size variations. Techniques that can be used in face preprocessing are adaptive histogram equalization, logarithm transform (LOG) and Gamma Intensity Correction (GIC) (Anila & Devarajan, 2012). The following subsection will describe these techniques.

#### Adaptive histogram equalization

It is a technique of enhancing image contrast. First, in a window around each pixel, histograms of grey levels (GLs) are created. To convert the input pixel GLs to output GLs, the cumulative distribution of GLs is used, which is the cumulative summation over the histogram. The output is maximally black if a pixel has a grey level (GL) less than all those in the surrounding window, and it is 50% grey if it has the median value in the window. An approximation $h$ of the local histogram is needed to equalize an input image $x$ with quantized GLs scaled between −1/2 and 1/2 where $m$ and $n$ are pixel’s coordinates. Any histograms are not actually evaluated by certain implementations but can be said to do so implicitly.

Using the Kronecker delta function $\delta (i,j)$ that is equal to one if $i = j$ and zero otherwise, we can begin with applying SIFT algorithm on those pixels in the input image with GL $g$. Spatial convolution with a rectangular kernel ${f_w}$ is used to find the number of such pixels in a window at each point. At the end, the estimate histogram sums to unity using an odd-integer value for a square window of width $w$ at each point (Stark, 2000). This can be written as:

(1) $$h(m,n,g) = \delta (g,x(m,n))\mathop {\rm \star }\limits^{m,n} {f_w}(m,n)$$

(2) $$f(m,n) = \left\{{\matrix{ {{w^{ - 2}},\ |m| \le (w - 1)/2,}  {|n| \le (w - 1)/2} \cr {\hskip-7pc}0,  {Otherwise} } } \right.$$

#### LOG

For grey scale transformations, LOG is a widely used approach. It simulates the human eye’s logarithmic sensitivity to light intensity (Anila & Devarajan, 2012). LOG allows us to present an image’s frequency content. A small range of low grey level values in the input image is converted to a larger range of output level values using this transformation. Higher input level values have the opposite effect. This transformation is used to extend the values of dark pixels in an image while compressing the higher level values. The log function has the essential property of compressing the dynamic range of images with large pixel value variations. The histogram of this data, on the other hand, is typically compact and uninformative (Hossain & Alsharif, 2007).

#### GIC

is a commonly used nonlinear intensity transformation function for manipulating contrast. The gamma correction can be written as,

(3) $$S = c{r^\gamma }$$

where $c$ denotes a constant, $r$ denotes a non-negative input pixel intensity, and $S$ is the post-processing output pixel intensity. The power factor (gamma) is used to generate a collection of curves for increasing different intensity zones. The transformation is known as an ${n^{th}}$ root transformation if the Gamma value is less than or equal to one. It is used to enhance darker intensity regions. The transformation curves are designed to improve brighter intensity regions if the Gamma value is greater than one, and the transformation is known as ${n^{th}}$ power transformation. As a result, depending on the value of Gamma, any range of intensity region can be improved (Acharya & Giri, 2020).

### Feature extraction

It is the stage at which features and relevant information are extracted and located for differentiating among different person faces in kinship. This step has a great effect on the classification performance accuracy. The performance reached by grouping face parts is much better than using the complete face (Ghahramani, Yau & Teoh, 2014). There are various techniques for extracting features that can be used for verification of kinship, such as histogram of oriented gradient (HOG), local binary patterns (LBP), compound local binary pattern (CLBP), Three-Patch LBP (TPLBP), Four-Patch LBP (FPLBP), scale invariant feature transform (SIFT), Gabor (Goyal & Meenpal, 2019; Zhu et al., 2014), grey-level co-occurrence matrix (GLCM), completed joint scale local binary pattern (CJLBP), Weber Local Descriptor (WLD) and Local Phase Quantization (LPQ). The following subsections provide description of of these techniques.

#### HOG

**Figure 4:**
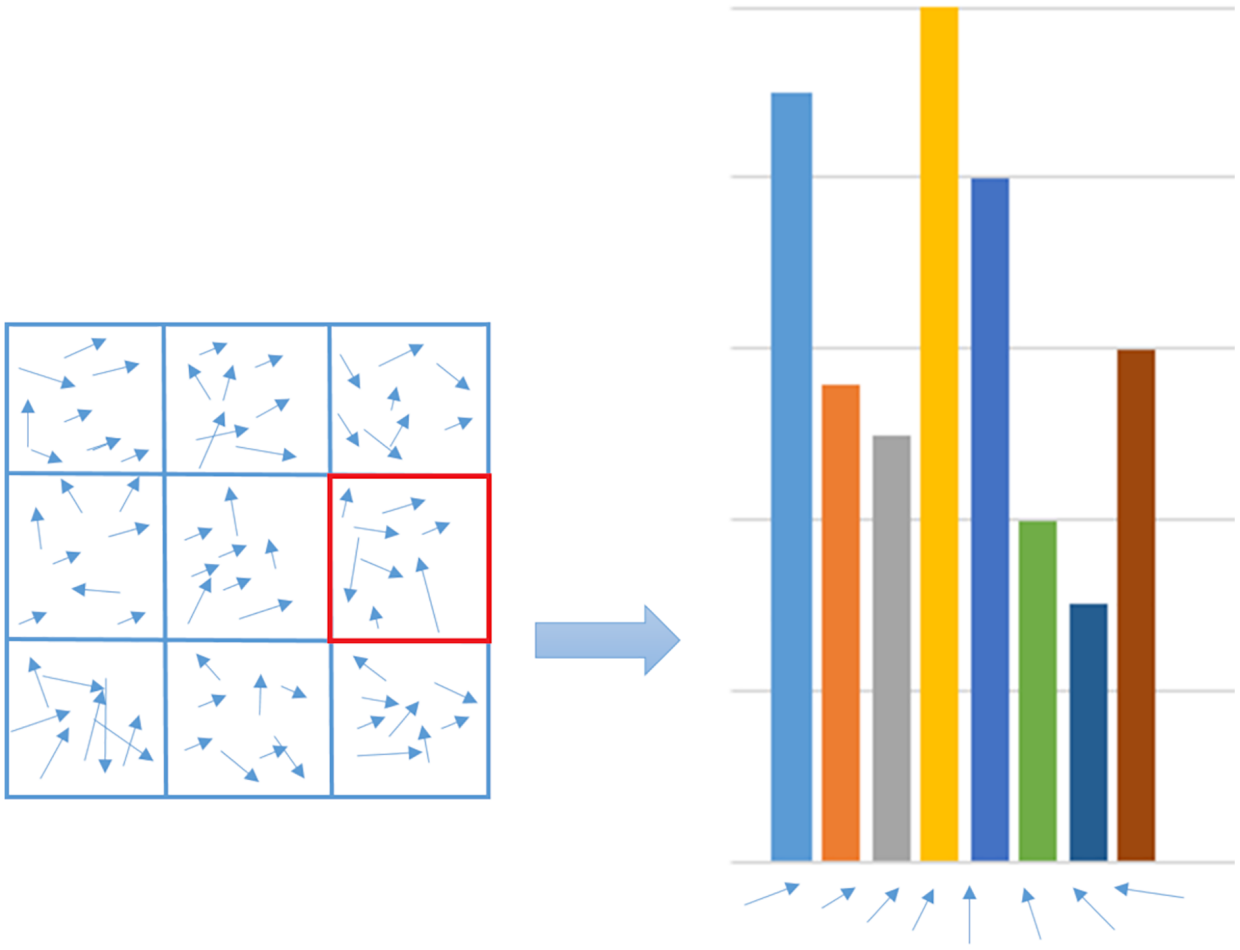
Histogram construction.

HOG is a feature descriptor that computes the number of gradient orientation occurrences in a detection window proposed for pedestrian detection (Déniz et al., 2011). Computing HOG can be summarized in three key steps. The first stage is computing gradient, where the spatial gradients are computed horizontally and vertically. The gradient of the image computes the intensity variation. Both of these gradients are used to calculate the magnitudes and angles of the gradient. The second stage is the binning of orientation, where the image is split into small related areas called cells. According to gradient angle, each pixel gradient magnitude in a cell is voted into various orientation bins. The last stage is describing the feature in which adjacent cells are combined into blocks in this stage (Cerna, Cámara-Chávez & Menotti, 2013). For each block, a histogram of gradient directions for the pixels within the cell is constructed as in Fig. 4. By its L2-norm, each block is normalized. In the detection window, the normalized block histograms are connected to make a descriptor. Mathematically, for a 2-D function $I(x,y)$, the gradients can be determined using the derivatives with respect to $x$ and $y$. Derivatives can be calculated by finite differences for a digital image where $x$ and $y$ are discrete values. The central difference-based technique is one of the ways used for computing image gradients. The gradient at pixel location $x,y$ can be written as:

(4) $${G_x} = I(x + 1,y) - I(x - 1,y),$$

(5) $${G_y} = I(x,y + 1) - I(x,y - 1)$$

where $Gx$ and $Gy$ refer to the horizontal and vertical gradients, respectively (Zhou et al., 2020).

#### LBP

**Figure 5:**
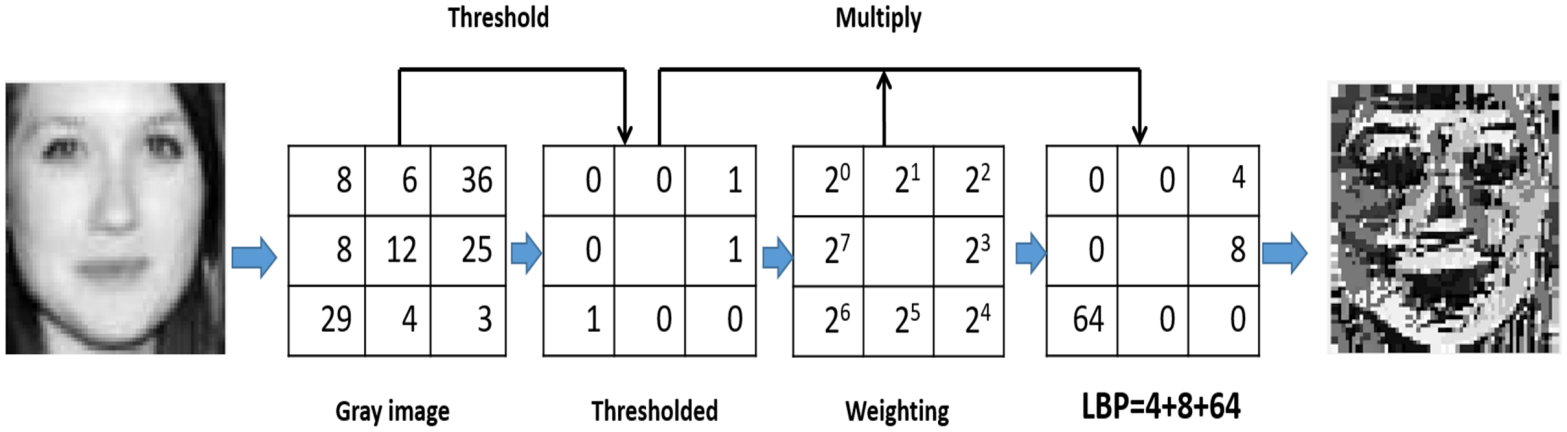
LBP code calculation.

It is an important invariant rotation method used for the extraction of texture features. LBP transforms grayscale image into encoded image (Mukherjee & Meenpal, 2019). It is one of the most popular descriptors since it is computed quickly and easily compared to the local and global descriptors proposed in the literature (Van & Hoang, 2019). Each central pixel is compared to its eight neighbours. Neighbours with a smaller value than the central pixel are assigned bit 0, while neighbours with a value equal to or greater than the central pixel are assigned bit 1. Thus, for each central pixel, a binary number can be generated by concatenating all of these binary bits in a clockwise direction. The generated binary number’s decimal value is used to replace the central pixel value (Moujahid & Dornaika, 2019; Kabbai et al., 2015; Ahonen, Hadid & Pietikainen, 2006; Ahonen, Hadid & Pietikäinen, 2004). A binary pattern representing texture characteristics will be generated by the threshold process as in Fig. 5 (the used image is provided from KinFaceW-I dataset dataset (Lu et al., 2013)). LBP basic equation can be written as:

(6) $$LBP({x_c},{y_c}) = \sum\limits_{n = 0}^7 {2^n}g({I_n} - I({x_c},{y_c}))$$

where $LBP({x_c},{y_c})$ is a LBP value at the centre pixel ${x_c},{y_c}$. ${I_n}$ is the value of neighbour pixel and $I({x_c},{y_c})$ is the centre pixel value. Index $n$ refers to neighbour pixels’ index. The function $g(x)$ = 0 if $x$ $<$ 0 and $g(x)$ = 1 if $x$ $\ge$ 0 (Prakasa, 2016).

#### CLBP

CLBP is an extension of LBP. It is proposed to solve LBP drawbacks as LBP is good at regular images only. LBP performance deteriorates in flat image cases, black spots, and bright image spots (Mukherjee & Meenpal, 2019). The LBP operator commonly fails to output acceptable binary code because it only considers the sign of the difference between two grey values. CLBP uses two bits for each neighbour, unlike LBP, which utilizes one bit for each neighbour to indicate simply the sign of the difference between the central and the corresponding neighbour grey values. The first bit, just like in the basic LBP pattern, represents the sign of the difference between the centre and the corresponding neighbour grey values, while the second bit encodes the magnitude of the difference in relation to a threshold value called the average magnitude ${M_{avg}}$. In the local neighbourhood of interest, ${M_{avg}}$ is the difference between the centre and neighbour grey values. If the magnitude of the difference between the centre and the corresponding neighbour is greater than the threshold ${M_{avg}}$, the CLBP operator sets this bit to 1. It is set to 0 otherwise as seen in the following Equation:

(7) $$S({i_p},{i_c}) = \left\{\matrix{ 00  {i_p} - {i_c} \lt 0,  |{i_p} - {i_c}| \le M_{avg} \\ 01  {i_p} - {i_c} \lt 0,  |{i_p} - {i_c}| \gt M_{avg} \\ 10  {i_p} - {i_c} \ge 0,  |{i_p} - {i_c}| \le M_{avg} \\ 11  Otherwise } \right.$$

where ${i_c}$ is the centre pixel’s grey value, ${i_p}$ is a neighbour p’s grey value, and ${M_{avg}}$ is the average magnitude of the difference between ${i_p}$ and ${i_c}$ in the local neighbourhood (Ahmed et al., 2011a, 2011b).

#### TPLBP

As the name says, TPLBP code is formed by comparing the values of three patches to produce a single bit value in the code supplied to each pixel. For each pixel in the image, a $w \times w$ patch centred on the pixel is examined and $S$ additional patches spread equally in a ring of radius $r$ around it. For a parameter $\alpha$, pairs of patches are taken, $\alpha$ patches apart along the circle, and compare their values with those of the central patch. A single bit’s value is determined by which of the two patches is closest to the central patch. $S$ bits per pixel are obtained as a result of this process. Specifically, we produce the Three-Patch LBP by applying the following formula to each pixel:

(8) $$TPLB{P_{\tau ,S,w,\alpha }}(p) = \sum\limits_i^S (f(d({C_i},{C_p}) - d({C_{i + \alpha\ {\rm mod}\ S}},{C_p}{))2^i}$$

where ${C_i}$ and ${C_{i + \alpha\ {\rm mod}\ S}}$ are two patches along the ring and ${C_p}$ is the central patch. The function *d*(,) is any distance function between two patches. $\tau$ value is slightly larger than zero (*e.g*., $\tau$ = 0.01) to provide some stability in uniform regions (Wolf, Hassner & Taigman, 2008).

#### FPLBP

Two circles of radius ${r_1}$ and ${r_2}$ based on each pixel in the image are used. $S$ patches of size $w \times w$ spread out uniformly on each circle for each pixel in the image. Two centre symmetric patches are compared in the inner ring with two centre symmetric patches in the outer circle positioned $\alpha$ patches distant along the circle to generate the Four-Patch LBP (FPLBP) code. Each pixel’s code contains a bit that indicates which of the two pairs being compared is more similar. As a result, we have $S/2$ centre symmetric pairs for $S$ patches along each circle, which is the length of the binary codes generated (Wolf, Hassner & Taigman, 2008). The FPLBP code has the following formal definition:

(9) $$TPLB{P_{{r_1},{r_2},S,w,\alpha }}(p) = \sum\limits_i^{S/2} f(d({C_{1i}},{C_{2,i + \alpha\ {\rm mod}\ S}}) - d({C_{1,i + S/2}},{C_{2,i + S/2 + \alpha\ {\rm mod}\ S}}{))2^i}$$

#### GLCM

GLCM is considered the second order statistics feature extraction method, which Haralick proposed in 1973 (Haralick, Shanmugam & Dinstein, 1973). Extraction is carried out using a co-occurrence matrix. Haralick suggested 14 features of GLCM that were transformed into 22 features currently. GLCM was a co-occurrence matrix on a grayscale image consisting of multiple lines and columns illustrating different grey levels $G$. The matrix element $G \times G$ stated a relative frequency where a certain angle and distance separated two pixels. There were 22 GLCM features, but 6 GLCM features can be covered here right now: contrast, correlation, homogeneity, second moment angular (ASM), energy, and entropy. Contrast results in measuring the difference of powers between a pixel and its neighbor overall the image. Correlation results in a measurement of how related a pixel are to its neighbor overall the image. ASM shows the summation of squared elements in the GLCM. Energy is the square root of ASM. Homogeneity results in a value that measures the closeness of the division of elements in the GLCM to the GLCM crosswise. Entropy is a scalar value that means the irregularity of grayscale images used to recognize the input image’s texture. The formulas of the six features are presented in Eqs. (6)–(11) (Babaghorbani et al., 2010; Wardhani et al., 2019).

(10) $$Contrast = \sum\limits_{i = 0}^{G - 1} \sum\limits_{j = 0}^{G - 1} P(i,j)(i - j{)^2}$$

(11) $$Correlation = \sum\limits_{i = 0}^{G - 1} \sum\limits_{j = 0}^{G - 1} P(i,j)\left[ {\displaystyle{{(i - {\mu _i}) - (j - {\mu _j})} \over {{\sigma _i} \times {\sigma _j}}}} \right]$$

$Where\ \mu = \displaystyle{{\sum_{i = 0}^{G - 1} \sum_{j = 0}^{G - 1} P(i,j)} \over {m \times n}},\sigma = \sqrt {\displaystyle{{\sum_{i = 0}^{G - 1} \sum_{j = 0}^{G - 1} {{(P(i,j) - \mu )}^2}} \over {m \times n}}}$

and $m \times n$ is the dimension of the image.

(12) $$Homogeneity = \sum\limits_{i = 0}^{G - 1} \sum\limits_{j = 0}^{G - 1} \displaystyle{{P(i,j)} \over {1 + {{(i - j)}^2}}}$$

(13) $$ASM = \sum\limits_{i = 0}^{G - 1} \sum\limits_{j = 0}^{G - 1} {(P(i,j))^2}$$

(14) $$Energy = \sqrt {ASM}$$

(15) $$Entropy = - \sum\limits_{i = 0}^{G - 1} \sum\limits_{j = 0}^{G - 1} P(i,j) \times \log \left[ {P(i,j)} \right]$$

#### SIFT

Lowe proposed the SIFT algorithm to extract the most stable interest points of an image and construct a vector descriptor using the Difference of Gaussian (DoG). The algorithm is described in the following steps Kabbai et al. (2015):

1. Scale-Space Extrema Detection

This step is responsible for constructing pyramids at various scales of the Gaussian function and interest points are extracted with invariant to scale and orientation.

**Figure 6:**
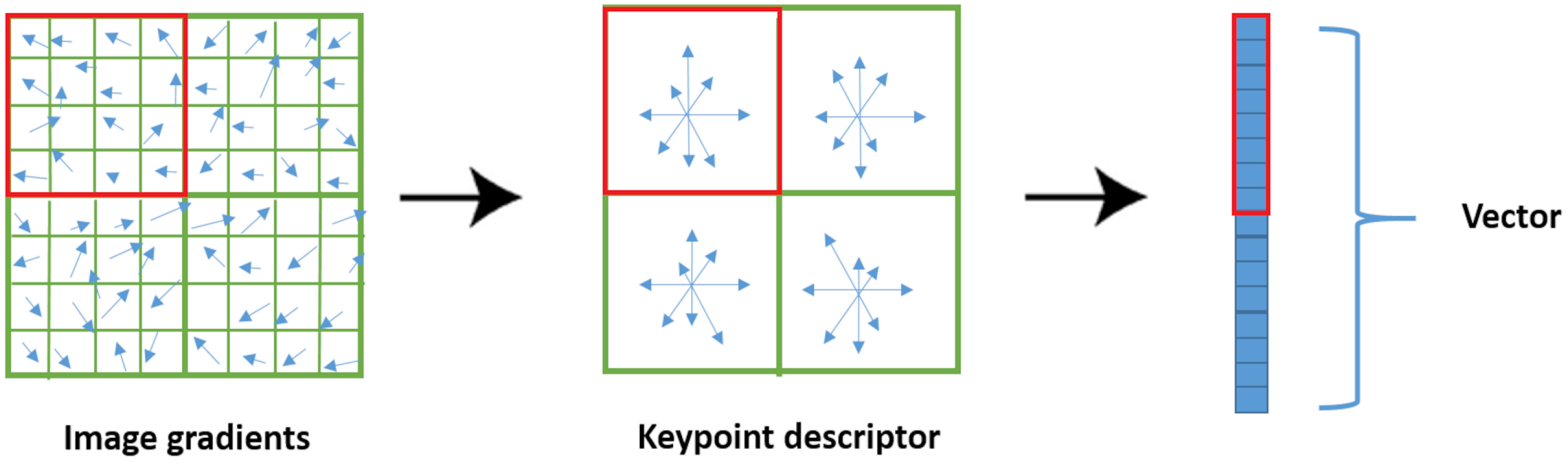
SIFT descriptor extraction.

2. Interest Point Localization
The previous stage yielded a significant number of candidates for interest points. To enhance the robustness of the interest points, this step includes discarding the interest points with poor contrast or poor localization along edges.
3. Orientation assignment
For each interest point detected in the preceding step, one or more orientations are assigned. This IP orientation constructs a histogram of gradient that includes 36 intervals.
4. Interest point descriptor
Lowe suggested that a set of histograms be created around an interest point in a 16-by-16-pixel window. 16 orientation histograms aligned in a 4 × 4 grid are used in typical interest point descriptors. Each histogram has eight orientations, resulting in a feature vector with 128 elements as seen in Fig. 6 (Kortli et al., 2018).

#### WLD

Based on Weber’s Law, it is a simple but effective and robust local descriptor. It is dependent on that human pattern perception is influenced not only by a stimulus’s change in intensity (such as sound or lighting), but also on the stimulus’s original intensity. Differential excitation and orientation are two components of WLD. The differential excitation is determined by the ratio of two terms: one is the current pixel relative intensity differences compared to its neighbours, and the other is the current pixel’s intensity. The gradient orientation of the current pixel is represented by the orientation variable (Chen et al., 2009).

#### LPQ

The LPQ texture analysis method uses the Fourier phase computed locally for a window in each image point. In eight-dimensional space, the phases of the four low-frequency coefficients are evenly quantized into one of 256 hypercubes that yield a 8-bit code. These LPQ codes are combined to form a histogram, which represents the texture and can be used to classify it (Ojansivu & Heikkilä, 2008).

### Feature selection

This stage is optional, which is dependent on the feature extraction phase. Its goal is to find the best collection of features that gives the best classification accuracy and lowest classification error. In the case of kinship, various studies used minimum redundancy maximum relevance (mRMR) (Dibeklioglu, Ali Salah & Gevers, 2013; Vieira, Bottino & Islam, 2013), Fisher’s score (Chergui et al., 2020; Moujahid & Dornaika, 2019) and Sequential Forward Selection (SFS) (Bottino, Vieira & Ul Islam, 2015; Kudo & Sklansky, 2000; Schenk, Kaiser & Rigoll, 2009).

### Kinship verification

After extracting absolute features from the two input facial images, this stage is responsible for classifying and checking whether the two images are kin or non-kin. This classification occurs by training the classifier using a labeled kin facial image dataset of kin. In preceding studies, many classifiers had been used in the verification process, like support vector machines (SVM) with different kernels (Vieira et al., 2014) and K-nearest-neighbor (KNN) (Goyal & Meenpal, 2019). The following subsections provide a brief description of these techniques.

#### KNN

The K-Nearest-Neighbours (KNN) is a non-parametric classification method that developed by Evelyn Fix and Joseph Hodges in 1951 (Fix & Hodges, 1952). It is a widespread technique of classification in data mining scenarios. It is a distance-based classifier. It has been widely used in many fields because of the implementation ease (Li & Cheng, 2009). A class membership is the result of kNN classification. A majority vote of its neighbours categorises an object, with the item being assigned to the most common class among its k closest neighbours (k is a positive integer, typically small). However, in order to employ kNN, a suitable value for k must be chosen, and the classification success is strongly dependent on this number. The data determines the best value for k; in general, larger values of k reduces the effect of noise on classification while blurring class boundaries. If *k* = 1, the item is simply assigned to that single nearest neighbor’s class. Euclidean or Manhattan distances can be used as a distance functions as seen in Eqs. (16) and (17), respectively.

(16) $$\sqrt {\sum\limits_{i = 1}^k {{({x_i} - {y_i})}^2}}$$

(17) $$\sum\limits_{i = 1}^k |{x_i} - {y_i}|$$

#### SVM

**Figure 7:**
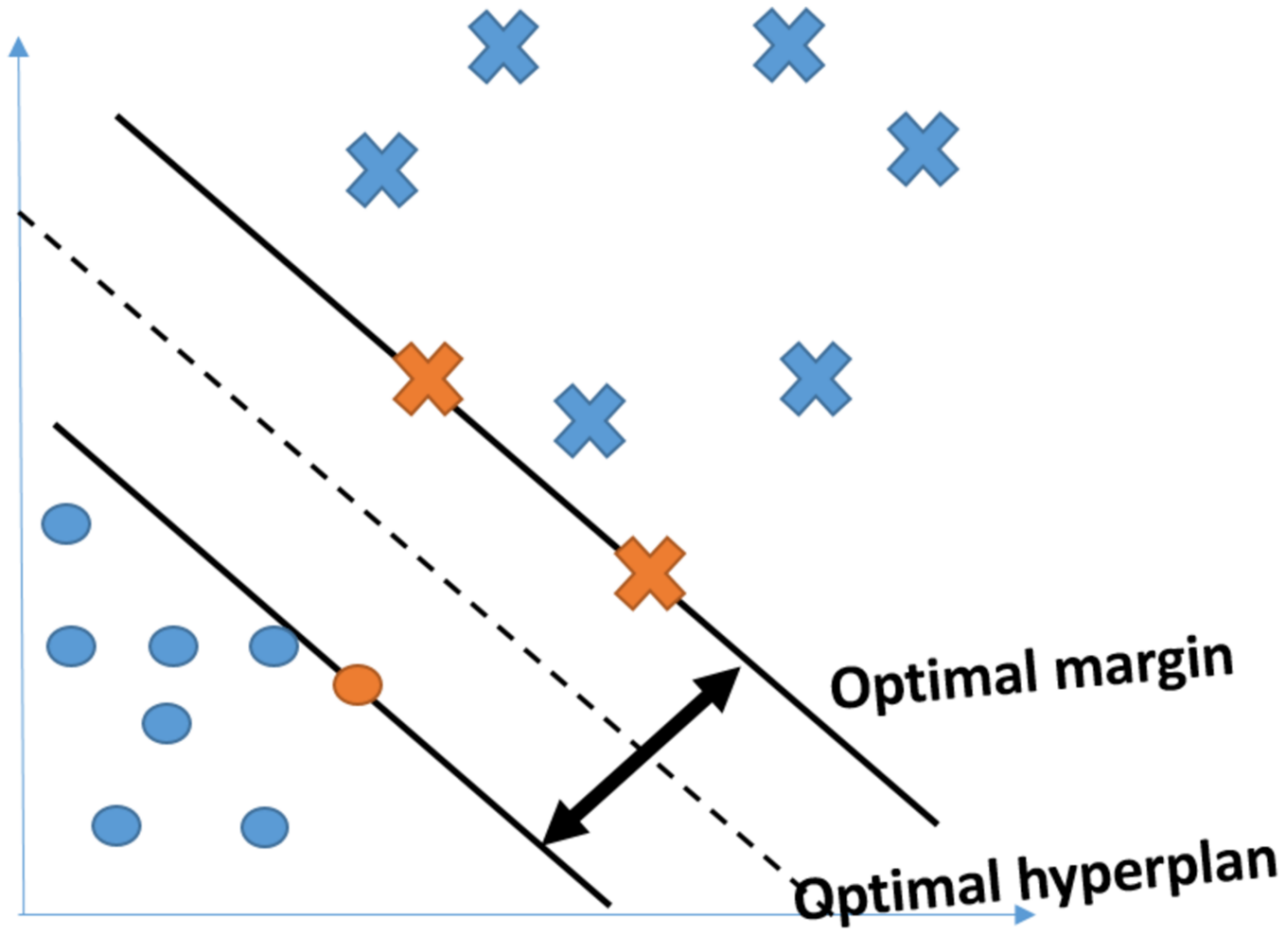
A separable problem in a two-dimensional space. The support vectors are represented by the orange color.

For classification and regression analysis, it is a supervised machine learning methodology. It’s sometimes referred to as an optimal margin classifier. It converts the input vectors into a high-dimensional feature space Z using a non-linear mapping. The data points nearest to the decision surface are called support vectors (or hyperplane). The data points that are the most difficult to categorise are these. They have a direct impact on the location of the decision surface. SVMs maximize the separating hyperplane’s margin (the’street’) as in Fig. 7 (Cortes & Vapnik, 1995).

## Benchmark datasets

Over the years, datasets have proven their main importance in computer vision research, allowing for targeted improvements and objective comparisons in various fields. In the case of kinship, many datasets can be used, such as families in the wild (FIW), CornellKinFace, UB KinFace, KinFaceW-I (KFW-I), KinFaceW-II (KFW-II), Tri-Subject Kinship Face (TSKinFace), and Family 101. The following subsections provide brief descriptions of these datasets.

### FIW

It is one of the primary largest datasets used for visual kinship identification. It involves complicated hierarchical relationships quickly and efficiently by just a small team offering the largest labeled group of up-to-date family images. With its complex label structure, FIW has been designed to serve multiple tasks. Within unconstrained environments, it has 13,000+ family images of thousand trees of families with 4-to-38 members captured in nature. FIW is structured as each family member has a distinctive identifier called family identifier (FID), and images gathered are allocated a distinctive identifier called photo identifier (PID). In the end, additional members have been allocated their distinctive identifier that is called member identifier (MID). For instance, FID1 → MID1 in PID1 means the first family member in the first collected image (Robinson et al., 2016, 2018).

### CornellKinFace

It is the first dataset that has been used for verifying kinship. The parent-child pair facial images database is gathered by a controlled online search for celebrities’ photos and their parents or children. Accordingly, it ended up having 150 pairs (300 images) with varying ages, gender, race, and career. For privacy issue, 7 families are taken out of the 150 families pairs. Kinship pairs’ distribution is 40% father-son (F-S), 22% father-daughter (F-D), 13% mother-son (M-S), and 25% mother-daughter (M-D) (Fang et al., 2010; Chergui et al., 2020).

### UB KinFace

“UB KinFace Ver1.0” is the primary dataset, including images of children and their parents at different ages. It consists of real-world public figures images (celebrities on social media, sports, and politicians) that have been downloaded from the web for kinship verification purposes. “UB KinFace” dataset Ver1.0 includes 270 images of 180 individuals that can be divided into 90 groups. Each group consists of child, young and old parent images. The idea is that facial images taken when they were young are more similar to those of their children than images taken when they are old (Xia, Shao & Fu, 2011).

“UB KinFace Ver2.0” is an expansion of "UB KinFace Ver1.0" if we take into account the impacts of more instances and ethnicity groups (Xia et al., 2012). It comprises six hundred images of four hundred people that can be split into two hundred categories. Depending on the race, ‘UB KinFace Ver2.0’ is categorized into two sections (*i.e*., Asian and non-Asian). Each of them comprises one hundred classes, two hundred people, and three hundred images. Four kin relationships typically exist (*i.e*., “F-S,” “F-D,” “M-S,” and “M-D”) (Shao, Xia & Fu, 2011).

### KFW-I and KFW-II

Both of them were created by searching the Internet, where face photos were captured under conditions that are uncontrolled in terms of illumination, clutter, facial expressions, and partial occlusion. As facial images in these datasets have been obtained from real-world environments, they are regarded as one of the largest facial datasets for verifying kinship for practical applications. KFW-I has four kin relationships: 156 F-S, 134 F-D, 116 M-S, and 127 M-D of family members. KFW-II dataset includes four kin relationships F-S, F-D, M-S, and M-D, 250 pairs of images for each kin relationship. The distinction between the two datasets is that each pair of kinship images in KFW-I was collected from separate images, while in KFW-II, each pair was collected from the same image. Consequently, KFW-II may minimize those limitations arising from the differences in illumination and aging in the KFW-I dataset (Lu et al., 2013).

### TSKinFace

Dataset images were collected by surfing the Internet depending on the awareness of common celebrities’ families and image networks, such as “flickr.com”. There are no restrictions enforced for posture, illumination, facial expression, background clutter, race, and photo quality. It contains two types of kin relationships as father-mother-son (FM-S) and father-mother-daughter (FM-D). The FM-S and the FM-D include 513 and 502 groups of tri-subject kin relationships, respectively. Totally, 1015 tri-subject groups are in the dataset. The families found in the dataset are different in terms of race. In the case of the FM-S relation, there are 343 and 170 tri-subject kin relationship groups for Asians and non-Asians, respectively. In the case of the FM-D relationship, the numbers for Asians and non-Asians groups are 331 and 171, respectively (Qin, Tan & Chen, 2015).

### Family 101

It contains 14,816 images with 101 various trees of families, containing 206 families and 607 persons. This dataset contains famous public families. It has four kinship relations 213 F-S, 147 F-D, 184 M-S, and 148 M-D (Fang et al., 2013).

## Performance measures

Before presenting the scientific efforts that proposed the automatic KVR system, it is important to mention the performance metrics of the KVR systems. There are multiple performance metrics, such as accuracy, precision, recall, and F1 score. These metrics depend on four terms true positive ( $TP$), false negative ( $FN$), false positive ( $FP$), and true negative ( $TN$) as indicated in Table 6, which represents the confusion matrix. It includes two classes, kin/non-kin, respectively. In kinship, $TP$ means the input image pair is kin, and the KVR system correctly classified them as kin. $FP$ means the input image pair are kin, and the KVR system incorrectly classified them as non-kin. $FN$ means the input image pairs are non-kin, and the KVR system incorrectly classified them as kin. $TN$ means the input image pairs are non-kin, and the KVR system correctly classified them as non-kin (Wardhani et al., 2019).

**Table 6:** Confusion matrix.

		Predicted	
		Kin	Non Kin
Actual	Kin	*TP*	*FP*
	Non Kin	*FN*	*TN*

Accuracy is an efficient performance indicator, which is represented by Eq. (18). It is essentially the proportion of samples that have been successfully classified to the total number of samples. Precision is the proportion of correctly classified kin samples to the overall samples that were classified as kin correctly or incorrectly, represented by Eq. (19). The recall (sensitivity) is the proportion of kin samples that were correctly classified to the total number of kin samples, represented by Eq. (20). The F1 score is the the harmonic mean of precision and recall, represented by Eq. (21) (Tharwat, 2020).

(18) $$Accuracy = (TP + TN)/(TP + FN + FP + TN)$$

(19) $$Precision = TP/(TP + FP)$$

(20) $$Recall = TP/(TP + FN)$$

(21) $$F1 = 2 \times \displaystyle{{Precision \times Recall} \over {Precision + Recall}}$$

## Literature review

Kinship verification is a very hot area of research that numerous studies have been addressed. In the following subsections, we will review the current kinship verification techniques, which do their best in extracting discriminating features. These techniques can be classified into the following three subsequent classes (Li et al., 2017): handcrafted feature-based, shallow metric feature-based, and deep learning feature-based techniques.

### Handcrafted feature-based techniques

Handcrafted techniques are dependent on the various methods of manual extraction of low-level features (Nanni, Ghidoni & Brahnam, 2017). These techniques are considered old kinship verification methods that always extract facial images handcrafted descriptors and use them for training classifiers. A summary of handcrafted feature-based techniques is in Table 7.

**Table 7:** A summary of some handcrafted feature-based methods.

Study	Features	Method	Datasets	Accuracy
Fang et al. (2010)	Color, Facial parts, Facial-distances, HOG	Facial feature extraction & forward selection methodology	150 public figures pairs	70.67%
Zhou et al. (2011)	SPLE	SPLE descriptor + SVM	400+ public figures pairs	67.75%
Zhou et al. (2012)	Gabor magnitude	GGOP	1,000 images	69.75%
Liu, Puthenputhussery & Liu, 2015	Color SIFT	IFVF	KinFaceW-I	73.45%
			KinFaceW-II	81.60%
Chergui et al. (2018)	LPQ, LDP	Multi-Level LDP and LPQ	CornellKinFace	82.86%
			UB KinFace	73.25%
			KinFaceW-I	75.98%
			KinFaceW-II	77.20%
Mukherjee & Meenpal (2019)	CLBP	CLBP and LFDA	KinFaceW-I	82.82%
			KinFaceW-II	89.36%
Chergui et al. (2019a)	LBP, BSIF, LPQ	Mixing different descriptors	CornellKinface	84.74%
			UB KinFace	82.89%
			Familly 101	81.69%
			KinFaceW-I	80.12%
			KinFaceW-II	78.16%
Goyal & Meenpal (2019)	HOG, LBP	Feature descriptors+ SVM classifier	KinFaceW-I	75.57%
Almuashi et al. (2021)	HOG	Feature learning & handcrafted features fusion	KinFaceW-I	68.6%
			KinFaceW-II	73.5%

Fang et al. (2010) proposed using modern methods for feature extraction, selection, and labeling face image pairs as “related” or “unrelated” in an intuitive way. They used a discriminative features list, such as hair color, eye color, skin color, and distance from nose to mouth, and merged them into a feature vector. They operated on a parent-child database of about 150 pairs of celebrities and public figures. They implemented KNN and SVM techniques to train a classifier on those various vectors and pairs of two unrelated people’s images. The accuracy of the proposed system was low.

Zhou et al. (2011) introduced an automated system for verifying kin relationships based on uncontrolled facial image analysis. Their research’s primary aim was to push verification of kinship from facial image research towards real scenarios. In replacement of being restricted to well-cropped faces with costly manual annotations, they concentrated on fully-automated verification of kinship from facial images under uncontrolled circumstances that place no constraints of posture, illumination, background, and expression. They used the spatial pyramid learning (SPLE) feature descriptor. They created a facial dataset by surfing the internet for more than four hundred pairs of public celebrities that were automatically detected with the Viola-Jones face detector.

Zhou et al. (2012) introduced a Gabor-based gradient orientation pyramid (GGOP) technique for representing features for verifying kinship from facial images under uncontrolled circumstances. To enhance multiple feature information, they proposed a new feature weighting technique to extract complementary information for verifying kinship. Experimental results had shown the effectiveness of their technique for kinship verification. Moreover, their proposed method’s performance was comparable to that of human observers.

Liu, Puthenputhussery & Liu (2015) presented an inheritable Fisher vector feature (IFVF) technique for verifying kin relationship. The Fisher vector was first extracted for every image by merging densely sampled SIFT features in opposing space of color. An inherited transformation that increased the similarity between kinship images while decreasing simultaneous learning for each image pair based on the Fisher vectors between non-kinship images was used. Consequently, the IFVF was exploited by seeking the inherited transformation on the Fisher vector for every image. In the end, they proposed a fractional power cosine similarity measurement that showed its theoretical roots in the Bayes decision rule for minimum error for verifying kin relationship. They worked on two datasets (KFW-I and KFW-II). Experimental findings indicated that their technique was capable of achieving good results compared to the existing techniques for verifying kinship.

Chergui et al. (2018) proposed that in order to verify kin relationship from face images, they used LPQ, local directional pattern (LDP), and multi-level (ML) descriptors. Their experimental findings showed that their technique produced a better performance than previous techniques. They operated on CornellKinFace, UB KinFace, KFW-I, and KFW-II datasets.

Mukherjee & Meenpal (2019) proposed a technique depending on CLBP and local feature-based discriminate analysis (LFDA) to enhance the accuracy of verifying kinship. These two techniques resulted in long feature vectors. Only the entire feature vector-based LFDA feature selection technique increased the compiler’s speed and chose the most suitable features. They used a KNN classifier. They worked on datasets of KFW-I and KFW-II.

Chergui et al. (2019a) introduced a technique depending on various mixed descriptors, such as LBP, LPQ, binary statistical image features (BSIF), and the multi-block (MB) representations. They also investigated the influence of various features for verifying kin relationships. Their technique was tested on five datasets, which are CornellKinFace, UB KinFace, Familly 101, KFW-I, and KFW-II.

Goyal & Meenpal (2019) proposed variable descriptors of features (LBP and HOG) to detect salient features. They also used an SVM classifier to gain knowledge of facial features extracted. They operated on the KFW-I dataset. Their findings indicated that the LBP-SVM approach outperformed HOG-SVM. The mean accuracy of the LBP-SVM technique was 75.57%. The mean accuracy of the HOG-SVM technique was 73.35%.

Almuashi et al. (2021) they presented a method, a fusion strategy made up of hand-crafted (low-level feature) and feature learning techniques (high-level feature) along with features that subtract the absolute value for the face pair. They applied HOG descriptor to extract hand-crafted features while CNN was used to represent the feature learning.

#### Shallow metric learning feature-based techniques

Researchers use metric learning techniques for learning discriminating features for verifying kinship to learn Mahalanobis distance depending on handcrafted features. This learning objective is making kinship pair similarity scores more than that of non-kinship pairs. A summary of shallow metric learning feature-based techniques is in Table 8.

**Table 8:** A summary of some shallow metric learning feature-based methods.

Study	Features	Method		Datasets
Lu et al. (2013)	LBP, LE, SIFT, TPLBP	NRML & MNRML	KinFaceW-I	69.9%
			KinFaceW-II	76.5%
Yan, Lu & Zhou (2014)	LBP, LBP+PDFL, LE, LE+PDFL, SIFT, SIFT+PDFL	PDFL+ multiview PDFL	KinFaceW-I	70.1%
			KinFaceW-II	77.0%
			CornellKinface	71.9%
			UB Kinface	67.3%
Yan et al. (2014)	LBP, SPLE, SIFT	DMML	KinFaceW-I	72.25%
			KinFaceW-II	78.25%
			CornellKinFace	73.63%
			UB KinFace	72.25%
Hu et al. (2014)	DSIFT, LBP, SSIFT, CSLBP, FPLBP, TPLBP, SIFT	$L{M^3}L$	LFW	89.57 *±* 1.53%
			YTF	81.28 *±* 1.17%
			KinFaceW-II	78.7%
Zhou et al. (2016)	LBP, HOG	ESL	KinFaceW-I	78.6%
			KinFaceW-II	75.7%
Zhao et al. (2018)	LBP, HOG, SIFT, LPQ, WLD	MKSM	KinFaceW-I	81.46%
			KinFaceW-II	82.45%
			TSKinFace	84.52%
			CornellKinFace	81.7%
Aliradi et al. (2018)	HoG, LBP	DIEDA	KinFaceW-I	80.60%
			KinFaceW-II	88.60%
			LFW	94.50 *±* 0.90%
Yan (2019)	D-CBFD	D-CBFD	KinFaceW-I	5.6%
Hu et al. (2019)	Facial feature points, SIFT	MvGMML	KinFaceW-	71.13%
			KinFaceW-II	69.70%

Lu et al. (2013) proposed a neighborhood repulsed metric learning (NRML) and multiview neighborhood repulsed metric learning (MNRML) methods for verification of kinship through facial image analysis by pulling intra-class samples near and pushing interclass samples far. They were looking forward to learning distance so that facial images with kinship relationships were set as near as possible. Those with no kinship relationships were pulled away as possible. They used several feature sets for face analysis in their work: LBP, LEarning-based (LE), scale invariant feature transform (SIFT), and TPLBP. This paper was the first trial to examine the verification of kinship on the large kinship datasets. They worked on two kinship face datasets KFW-I and KFW-II. Experimental findings showed that their approaches were better than previous metric learning techniques and were better than human observers in verifying kinship in facial images at that time.

Yan, Lu & Zhou (2014) presented prototype-based discriminative feature learning (PDFL) and multiview PDFL (MPDFL) techniques for verifying kin relationships. They constructed a set of face samples with unlabeled kin relations from the labeled face in the wild dataset as the reference set. After that, every sample was defined as a mid-level feature vector in the training facial kinship database. They worked on four databases KFW-I, KFW-II, UB KinFace, and CornellKinFace.

Yan et al. (2014) introduced discriminative multimetric learning (DMML) technique for verifying kin relationships by using facial images. Given each facial image, they first extracted many features using various facial descriptors LBP, SPLE and SIFT to analyze facial images from various aspects. Various feature descriptors can give supportive information. After that, they learned several distance measurements with these derived features. The likelihood of a pair of facial images with a kin relationship having a smaller distance than that of the non-kin pair’s relationship is increased. They operated on KFW-I, KFW-II, UB KinFace, and CornellKinFace datasets.

Hu et al. (2014) presented a large margin multimetric learning ( $L{M^3}L$) technique for verifying kin relationship that jointly learned several measurements of distance that could be used to maximize the correlations of each sample’s several feature representations. Each positive pair’s distance was less than a low threshold, and that of each negative pair is greater than a high one. Their paper worked on labeled faces in the wild (LFW) (Huang et al., 2008), YouTube faces (YTF) (Wolf, Hassner & Maoz, 2011), and KFW-II datasets. On the LFW dataset, they extracted three different features, which are dense SIFT (DSIFT) (Lowe, 2004), LBP, and sparse SIFT (SSIFT). On YTF, they used center-symmetric LBP (CSLBP) (Wolf, Hassner & Maoz, 2011) and four-patch LBP (FPLBP) feature descriptors. On KFW-II, four types of features, which are LE (Cao et al., 2010), LBP, TPLBP, and SIFT, were extracted.

Zhou et al. (2016) introduced an ensemble similarity learning (ESL) technique for verifying kin relationship. They operated on KFW-I and KFW-II datasets. They applied HOG and LBP for feature extraction. Experimental results indicated that their technique was superior to previous techniques according to both the verification rate and computation efficiency.

Zhao et al. (2018) proposed a multiple kernel similarity metric (MKSM). It was different from the Mahalanobis metric as the similarity calculation depended on an implicit nonlinear feature transformation. Total MKSM was a weighted aggregation of main similarities, which controlled the capacity for fusing features. The key similarities were exploited from basic kernels, local features, and weights calculated by solving a restricted linear programming (LP) problem resulting from a large margin (LM) criterion. Tests on KFW-I, KFW-II, CornellKinFace, and TSKinFace datasets demonstrated a comparable performance with previous techniques.

Aliradi et al. (2018) proposed a framework that used discriminating information depending on the exponential discriminant analysis (DIEDA) associated with various scale descriptors. Features were exploited by LBP and HOG feature extraction techniques. They presented a system of verification dependent on integrating two different kinds of feature extraction in various scales. Their approach learned to decide whether two individuals were in a kinship or not. They used a side-information-based linear discriminant analysis (SILD) to learn a distance metric. They worked on three datasets which were KFW-I, KFW-II, and LFW datasets (Huang et al., 2008).

Yan (2019) presented a discriminative compact binary face descriptor (D-CBFD) for verifying kin relationship. They calculated pixel difference vectors (PDVs) on local patches with a facial image first. Then, they learned a discriminating projection to transform each PDV into a low-dimensional binary space of features. In the end, they pooled these binary feature vectors into a histogram function within each face. To illustrate their methodology’s efficacy, they operated on three kinship databases KFW-I, KFW-II, and kinship face videos in the wild (KVFW) (Yan & Hu, 2018).

Hu et al. (2019) presented a multiview geometric mean metric learning (MvGMML) technique for verifying kinship using facial images in the real-world. They presented a practical misalignment-robust kinship verification framework. They localized nine facial features using a facial feature detector. For each localized facial feature point, they extracted a dense SIFT descriptor around the feature point. Experimental findings on two datasets (KFW-I and KFW-II) demonstrated that the efficacy of their technique.

#### Deep learning feature-based techniques

Deep learning is widely used in representing and classifying images. Increasingly, papers adopt deep learning approaches to learn discriminating features for verifying kinship (Li et al., 2017). A summary of deep learning feature-based techniques is in Table 9.

**Table 9:** A summary of some deep learning feature-based methods.

Study	Deep network	Method	Datasets	Accuracy
Dehghan et al. (2014)	Autoencoder	Discriminative Neural network features fusion	KinFaceW-I	74.5%
			KinFaceW-II	82.2%
Wang et al. (2015)	Stacked Auto-encoder Network	DKV	KinFaceW-I	66.9%
			KinFaceW-II	71.3%
Zhang et al. (2015)	CNN	CNN based on key points	KinFaceW-I	77.5%
			KinFaceW-II	88.4%
Kohli et al. (2016)	fcDBN	KVRL-fcDBN	CornellKinface	89.5%
			UB kinFace	91.8%
			KinFaceW-I	96.1%
			KinFaceW-II	96.2%
			WVU	90.8%
Duan, Zhang & Zuo (2017)	cCCN	CFT	KinFaceW-I	77.4%
	fCCN		KinFaceW-II	79.3%
			CornellKinFace	78.6%
			UB KinFace	72.3%
Lelis (2018)	FaceNet	DLML	KinFaceW-I	70.20%
			KinFaceW-II	66.72%
			UB KinFace	67.38%
Chergui et al. (2019b)	VGG-Face	Face preprocessing, Features extraction, normalization, and selection, Kinship verification	CornellKinFace	92.74%
			UB KinFace	90.39%
			Familly 101	86.49%
			KinFaceW-I	79.37%
			KinFaceW-II	84.67%
Nandy & Mondal (2019)	SqueezeNet	Approach using Siamese CNN + cosine similarity	FIW	67.66%
Dornaika, Arganda-Carreras & Serradilla (2020)	VGG-F	Deep features fusion using CNN	KinFaceW-I	66.85%
	VGG-Face		KinFaceW-II	77.35%
Chergui et al. (2020)	VGG-Face	VGG-face	CornellKinFace	92.89%
		CNN Model	UB KinFace	90.59%
			Familly 101	84.82%
			KinFaceW-I	86.65%
			KinFaceW-II	81.11%
Yan & Song (2021)	CNN	Deep relational network	KinFaceW-I	85.6%
			KinFaceW-II	88.8%
Almuashi et al. (2021)	CNN	Feature learning & handcrafted features fusion	KinFaceW-I	68.6%
			KinFaceW-II	73.5%

Dehghan et al. (2014) illustrated that features and metrics could be fused through gated auto-encoders to learn essential features representing parent-offspring similarities. They developed a novel approach for learning discriminating and genetic features to explain the relationship between parent and offspring. This approach uncovered three main insights that reduce the difference between anthropological research and computer vision. First, they found that their findings corroborated that offspring resemble their parents with a probability higher than chance. Second, they concluded that a daughter resembles her mother more often than her father while a son only slightly favors the father. Third, their algorithm extracted features that looked like those found in anthropological research—for example, the eyes and nose’s parts.

Wang et al. (2015) introduced a deep kinship verification (DKV) technique by deep learning framework integration with metric learning to choose nonlinear features. Their technique wasn’t like current shallow techniques that depend on metric learning for verifying kin relationships. It could discover the required project space to make sure the largest margin of negative sample pairs (*i.e*., parent and child with non-kin relationship) and the smallest margin of positive sample pairs. Their experimental findings showed that their approach achieved good performance on KFW-I and KFW-II datasets.

Zhang et al. (2015) presented deep convolutional neural networks (CNNs) to verify kinship using facial image analysis. The proposed approach generated high-level features associated with key-points-based representations. Experimental findings demonstrated that their approach largely enhanced the performance of the existing technique and outperformed human performance. On two kinship datasets KFW-I and KFW-II, their method achieved 5.2% and 10.1% improvements compared to the previous techniques, respectively.

Kohli et al. (2016) introduced a technique for representing features known as filtered contractive deep belief networks (fcDBN). A hierarchical kinship verification *via* representation learning (KVRL) framework is used to learn the representation of various face regions in an unsupervised manner. Using filters and contractive regularization penalty, proposed feature representation encoded relational information appeared in the images. As an output from the learned technique, a facial image kin representation was exploited, and a multi-layer neural network was used to check the kin properly. To simplify kinship verification, a WVU kinship dataset composed of multiple images of persons was created. The results showed the WVU kinship dataset and four existing datasets (KFW-I, KFW-II, UB KinFace, and CornellKinFace), their system (KVRL-fcDBN) outperformed the current kinship verification techniques accuracy.

Duan, Zhang & Zuo (2017) presented a coarse-to-fine transfer learning (CFT) technique for verifying kin relationships. The technique was motivated by the transferable value of facial knowledge and the kinship data scarcity issue in training a deep model. By using other large-scale domain data, they tried to explore the learning issue of small-scale data. Specifically, two deep CNN models, including coarse CNN (cCNN) and fine CNN (fCNN), were used in CFT to capture specific semantic features of high-level and discriminating kin-relation. They operated on KinFaceW-I, KinfaceW-II, CornellkinFace, and UB kinFace datasets.

Lelis (2018) introduced a solution to the problem of kinship verification using a non-context-aware approach deep linear metric learning (DLML). The approach was validated using a large age variation dataset such as UB KinFace. The technique leverages multiple deep learning architectures trained with massive facial databases. Additional tests were also performed on the KFW-I and KFW-II datasets for further assessment of their technique.

Chergui et al. (2019b) proposed a technique that took two images as an input and then generated (kin/non-kin) as an output. The technique included five stages: face preprocessing, extracting deep features, representing and normalizing pair features, selecting features, and verifying kinship. Experiments were conducted on five public datasets, which are CornellKinFace, UB KinFace, Family 101, KFW-I, and KFW-II. The experimental findings showed that their technique was comparable with existed techniques.

Nandy & Mondal (2019) introduced a deep learning technique using siamese convolutional neural network architecture to compute the similarity between two given images. They used two parallel SqueezeNet networks. The networks had initial weights from training the SqueezeNet on the VGGFace2 Dataset (Cao et al., 2018). Then, they further trained this architecture on the FIW dataset (Robinson et al., 2018, 2017, 2016). They used similarity metrics and completely connected networks to connect the two networks to one output.

Dornaika, Arganda-Carreras & Serradilla, 2020 proposed an approach that extracted deep facial features for verifying kinship. A robust feature selection and discriminant projection of kinship-oriented data were incorporated into the structure. The system included three stages of fusion (1) early fusion of descriptors where the selection of filters selected the most important deep features, (2) a middle-stage fusion using a kinship-based multiview metric learning technique, and (3) a late-stage fusion that integrated the responses from the classifiers. The pre-trained deep convolution neural networks VGG-F and VGG-Face were provided with face features that were mainly proposed for groups of objects and identities discrimination. Experimental findings on two datasets (KFW-I and KFW-II) revealed that without using external data or data augmentation used to validate kinship, the proposed system outperformed the best existing techniques.

Chergui et al. (2020) proposed a technique of kinship verification depending on CNN Model (VGG-Face). They used the Fisher score for selecting features and SVM for query relationship between the son and the father’s images. Their findings were good and comparable with other methodologies. They suggested that their results could be enhanced in the future using other convolution-neural-network methods like Inception ResNet-v2, Inception-v4, and VGG-Face2.

Yan & Song (2021) presented a deep relational network that used multi-scale information from facial images. Their method combined two CNNs with shared parameters for each pair of face images to extract distinct scales of features. Their network made use of the benefits of convolutional computations to turn distinct parts of a face into different scale features at the same time. Multi-scale features were employed to supply the network with global and contextual information.

As we have discussed the KVR stages, kinship benchmark datasets and the kinship performance measures in the previous sections, it is necessary to discuss the limitations that affect the kinship verification performance. Hence, the next section provides a detailed description of these limitations.

## Current limitations

As previously discussed, many limitations threaten the automatic KVR system. These limitations strongly affect the accuracy of the system. Limitations can be classified into two following categories: database and kinship itself limitations.

### Database limitations

Many limitations can affect both KVR and face recognition systems database images and lead to bad accuracy, such as illumination, occlusion, facial expressions, pose variation, clutter, and low resolution.

#### Illumination

**Figure 8:**
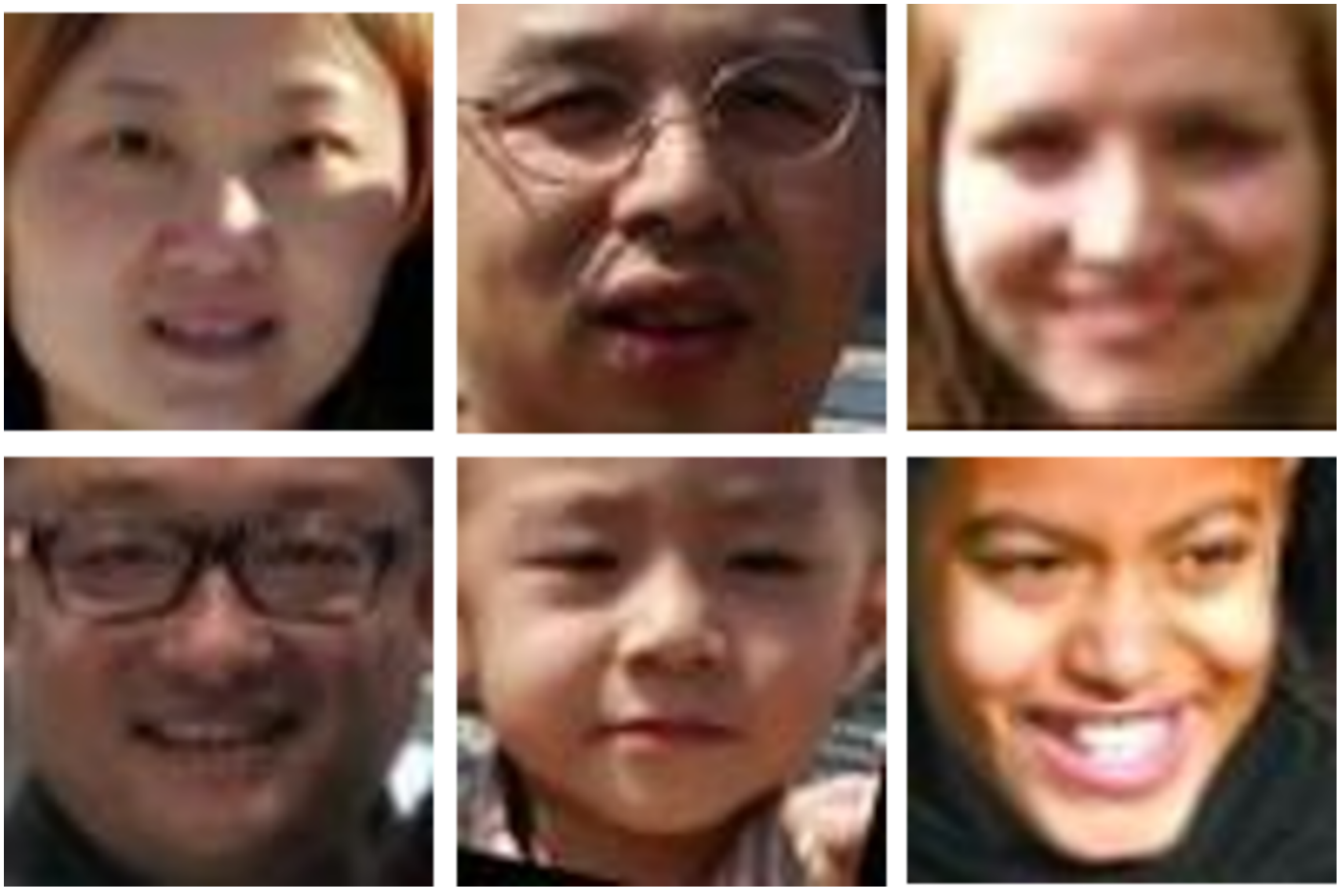
Illumination variations (Lu et al., 2014, 2012).

Illumination means variations of light. The total value of light intensity reflected from an object and the shading pattern and shadows seen in an image can vary with illumination changes. Nonetheless, illumination variations can output more extensive image changes than changing either the identity or the face viewpoint. The same photograph with the same camera position and captured with the same facial expression and posture may look totally different with the lighting conditions changes. Face recognition problem related to illumination changes is widely identified to be hard for humans and techniques. Therefore, the challenges presented by varying lighting situations remain a significant issue for automatic face identification systems. It is observed that the difference between two images taken under different illumination of the same person is greater than the distinction between the images captured under the same illumination conditions of two different persons. Variation of light changes the appearance of the face extremely, as shown in Fig. 8 (all images are provided from KinFaceW-I dataset (Lu et al., 2013)) (Ohlyan, Sangwan & Ahuja, 2013; Liu, Lam & Shen, 2005).

#### Occlusion

**Figure 9:**
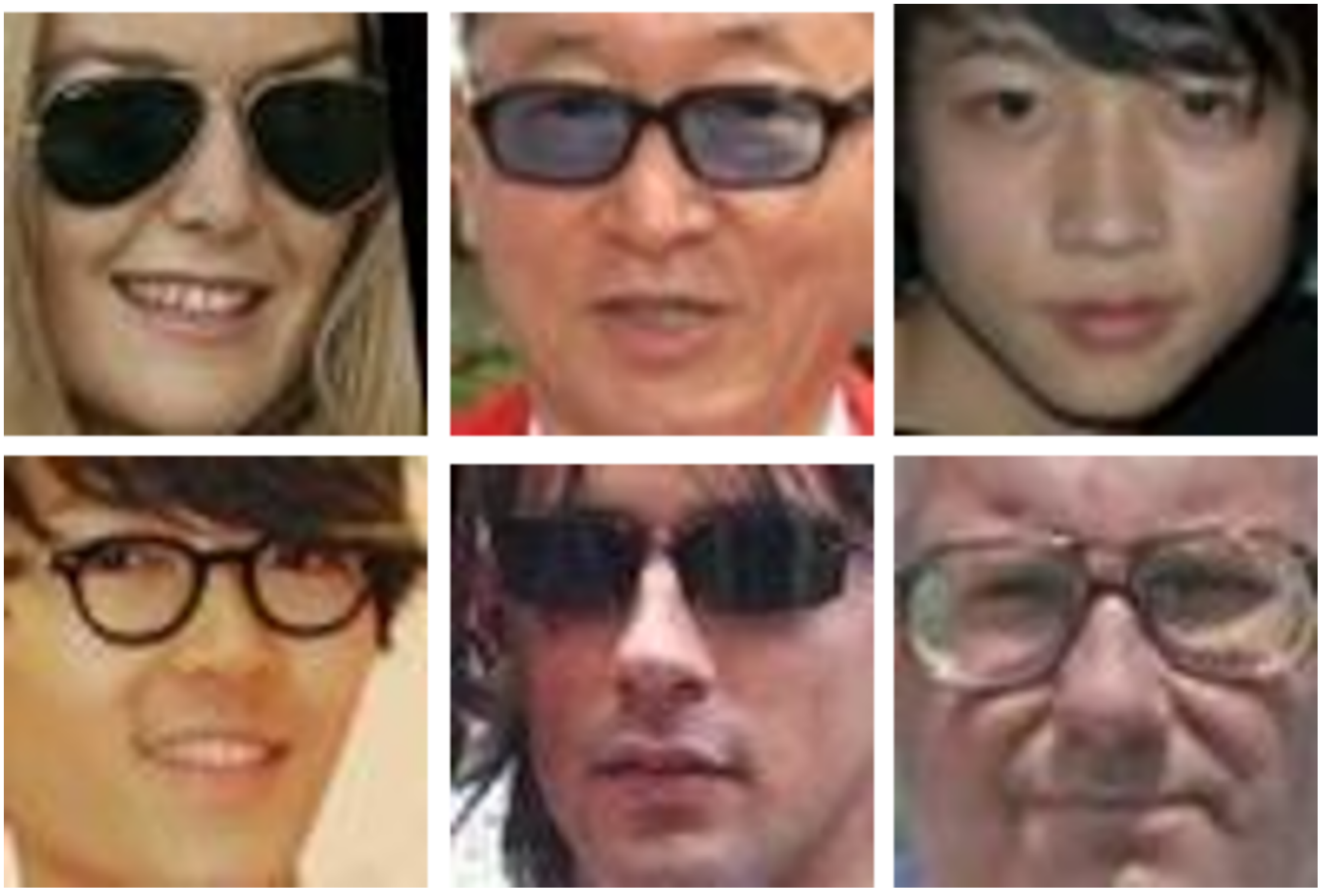
Example of some occluded facial images (Lu et al., 2014, 2012).

One of the most difficult and challenging issues for KVR is partial face occlusion. In real-world applications, a face identification system may encounter many occluded faces due to using accessories such as sunglasses, scarf, or hands on the face, as shown in Fig. 9 (all images are provided from KinFaceW-I dataset (Lu et al., 2013)). Furthermore, the things that persons hold and external sources block the camera view partially. According to that, the facial systems must be invariant to the occlusion to guarantee operation in a reliable real-world (Ekenel & Stiefelhagen, 2009; Priya & Banu, 2014).

#### Facial expressions

**Figure 10:**
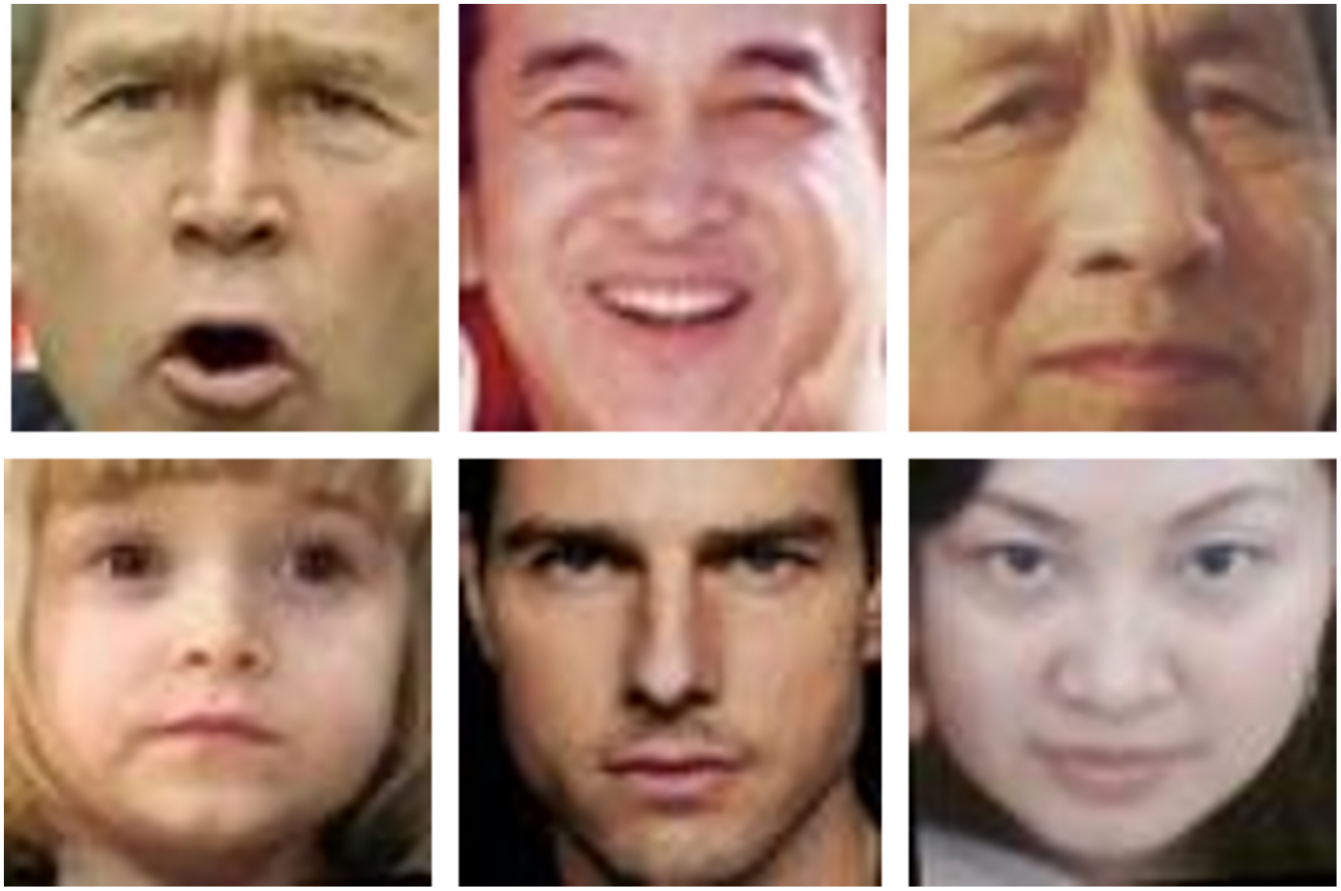
Expression variation (Lu et al., 2014, 2012).

The face is a vital bio-metric of humans, which has a great function in recognizing human identity and modes because of its unique features. Human expression changes due to his/her emotions, which appear in various facial expressions, as shown in Fig. 10 (all images are provided from KinFaceW-I dataset (Lu et al., 2013)). These variations in facial expressions affect the appearance that, in turn, makes it hard for facial recognition systems to match the exact face stored in the database (Ohlyan, Sangwan & Ahuja, 2013).

#### Pose variations

**Figure 11:**
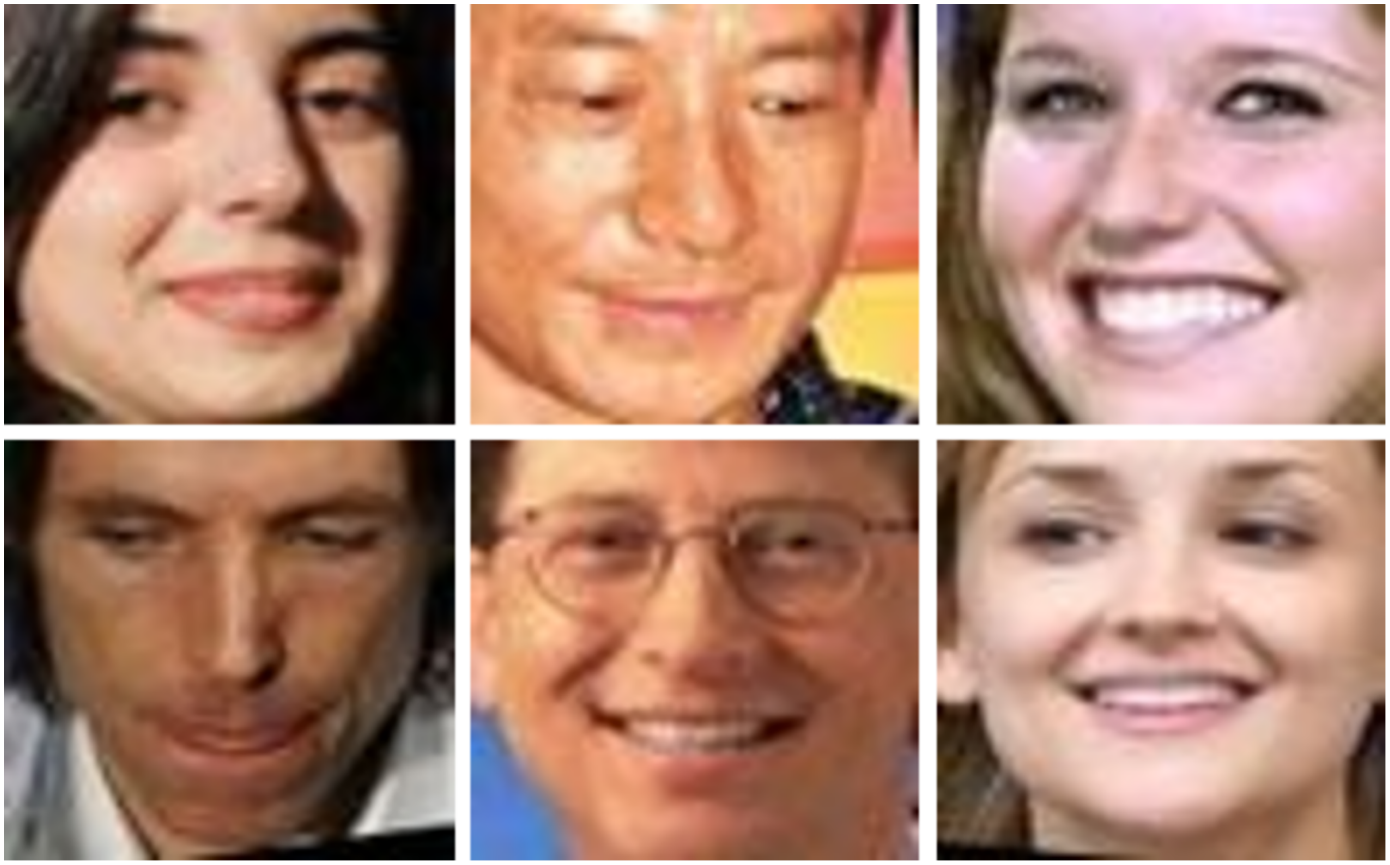
Pose variation (Lu et al., 2014, 2012).

In face recognition, posture variations in images are also a significant limitation. Face posture varies with the observer’s angle of view, position, and rotation of the head, as shown in Fig. 11 (all images are provided from KinFaceW-I dataset (Lu et al., 2013)). Posture variations explode, a severe challenge for the recognition of the input image. A face recognition system can be accepted in small rotation angles, but it becomes a problem when rotation increases. This leads to wrong identification or no recognition (Ohlyan, Sangwan & Ahuja, 2013; Gao et al., 2007).

#### Low resolution

It measures image quality as the face recognition algorithms cannot give acceptable performance on a very low resolution (VLR) facial image (Zou & Yuen, 2011). Low-resolution challenges appear in a facial system when the facial image resolution is less than 16 × 16. Many monitoring applications, such as small-scale standalone camera programmes in supermarkets and banks, have this issue. Where images are usually taken from a surveillance camera consisting of a very small face area and can’t support adequate face resolution for identification, the face area would be smaller than 16 × 16 when the person’s face is far away from the camera. There are few features in such a low-resolution face image, so most of the details can be lost. This can lead to a significant deterioration in identification efficiency (Ohlyan, Sangwan & Ahuja, 2013).

#### Clutter

Automated detection of persons’ faces from images with clutter problems is critical for facial recognition and security applications. This challenge is complex due to the multiple differences and the ambiguity between facial and background areas (Huang et al., 2003). This means there are many objects in the image, and it is hard for an observer to concentrate on a single object. Several persons in the image will find it difficult to detect each one.

#### Noise

It means that pixels display different intensity values in an image instead of real pixel values obtained from the image. Noise typically contaminates the databases gathered by image sensors. Imperfect tools, data collection problems, and blocking natural phenomena can all reduce the required data. In addition, noise is caused by transmission and compression errors (Motwani et al., 2004). Noise can influence segmentation, feature extraction, and image recognition processes. Accordingly, the effect of noise on face identification is an interesting field of pattern recognition (Zhao, Wang & Zhang, 2014).

### Kinship itself limitations

The second category of the limitations that can affect the KVR system is the one that is directly related to kinship itself. This category includes variation in age, gender, and verification feature resemblance between kin. The following subsections present a brief description of kinship itself limitations.

#### Variation in age

**Figure 12:**
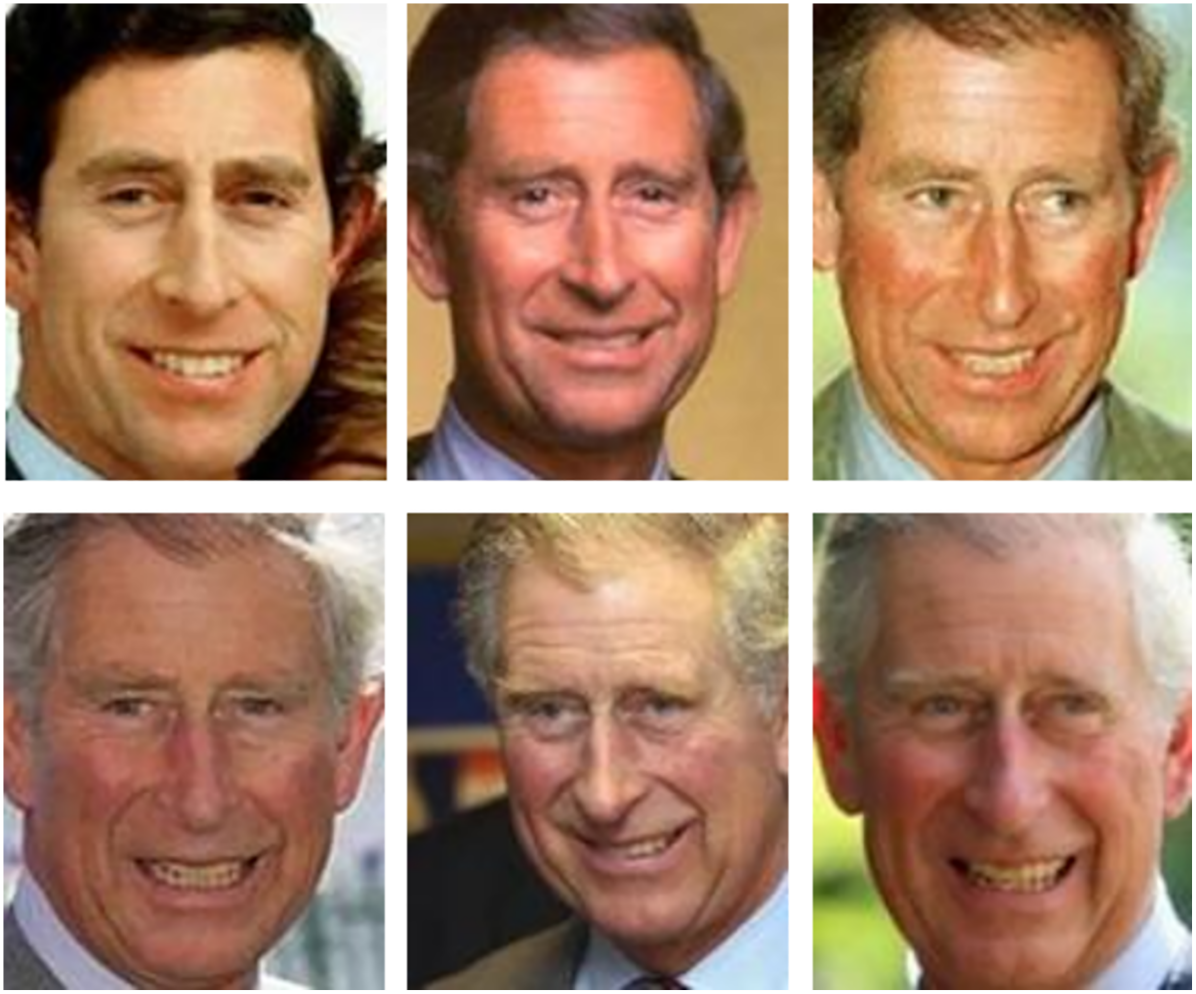
Age variation (Ruogu et al., 2013).

The face features are deeply affected over the years. Since the old parent’s face features change when matched to their face when they were small, this age variation maximizes the gap between the child-old parent’s face, making it hard to verify the kin relationship (Lelis, 2018). Figure 12 (the images are provided from Family 101 dataset (Fang et al., 2013)) shows how person’s face changes over the years.

#### Variation in gender

Also, gender is one of the challenges that affect kinship verification accuracy. Kinship accuracy of father-daughter is less than mother-daughter, and father-son accuracy is higher than mother-son, so gender greatly affects the verification accuracy.

#### Verifying feature resemblance between Kin

**Figure 13:**
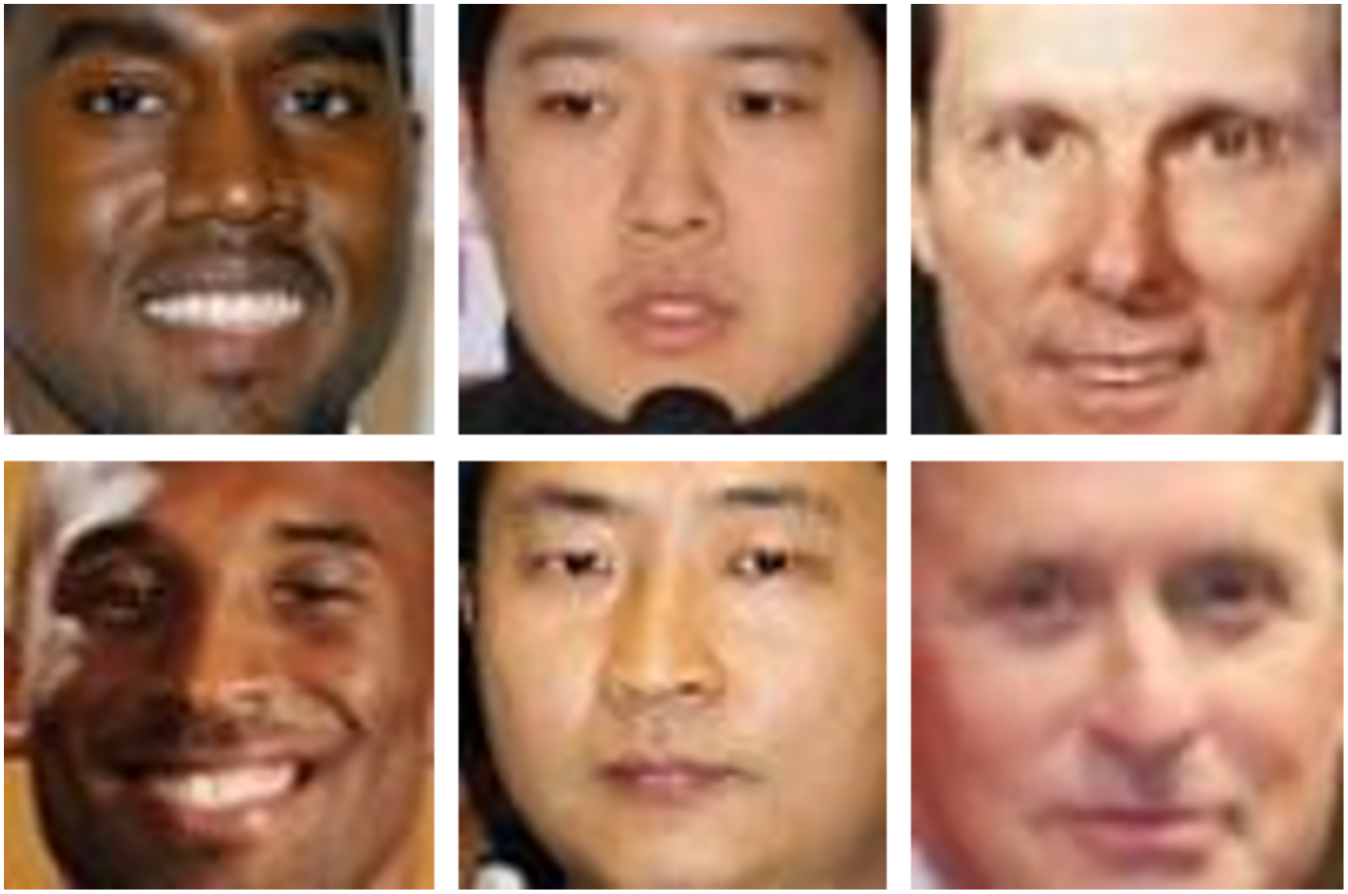
Photos with high appearance similarity but similar features are low (Lu et al., 2014, 2012).

Means distinctive extraction of features. Feature extraction can be thought of as very sensitive in verifying and recognizing kinship. For example, two children from the same parent and different mothers or same mother and different parents will be difficult to get distinctive features as children from the same parent and mother may inherit the same feature in different ways. Another example, we may have two images in which the appearance is high, but features are low (Almuashi et al., 2017). People with dissimilar appearances can be in kinship relationships, and people with similar appearances can be in non-kin relationships as shown in Fig. 13 (all images are provided from KinFaceW-I dataset (Lu et al., 2013)) (Harpending, 2002; Lelis, 2018).

## Future directions

Kinship topic is expected to attract growing interest from many fields, such as computer vision, pattern recognition, and machine learning, because of its difficulty and extensive functional applications. Despite many good works and trials on kinship verification issues, there are still various promising research directions that could be suggested (Qin, Liu & Wang, 2020).

**Figure 14:**
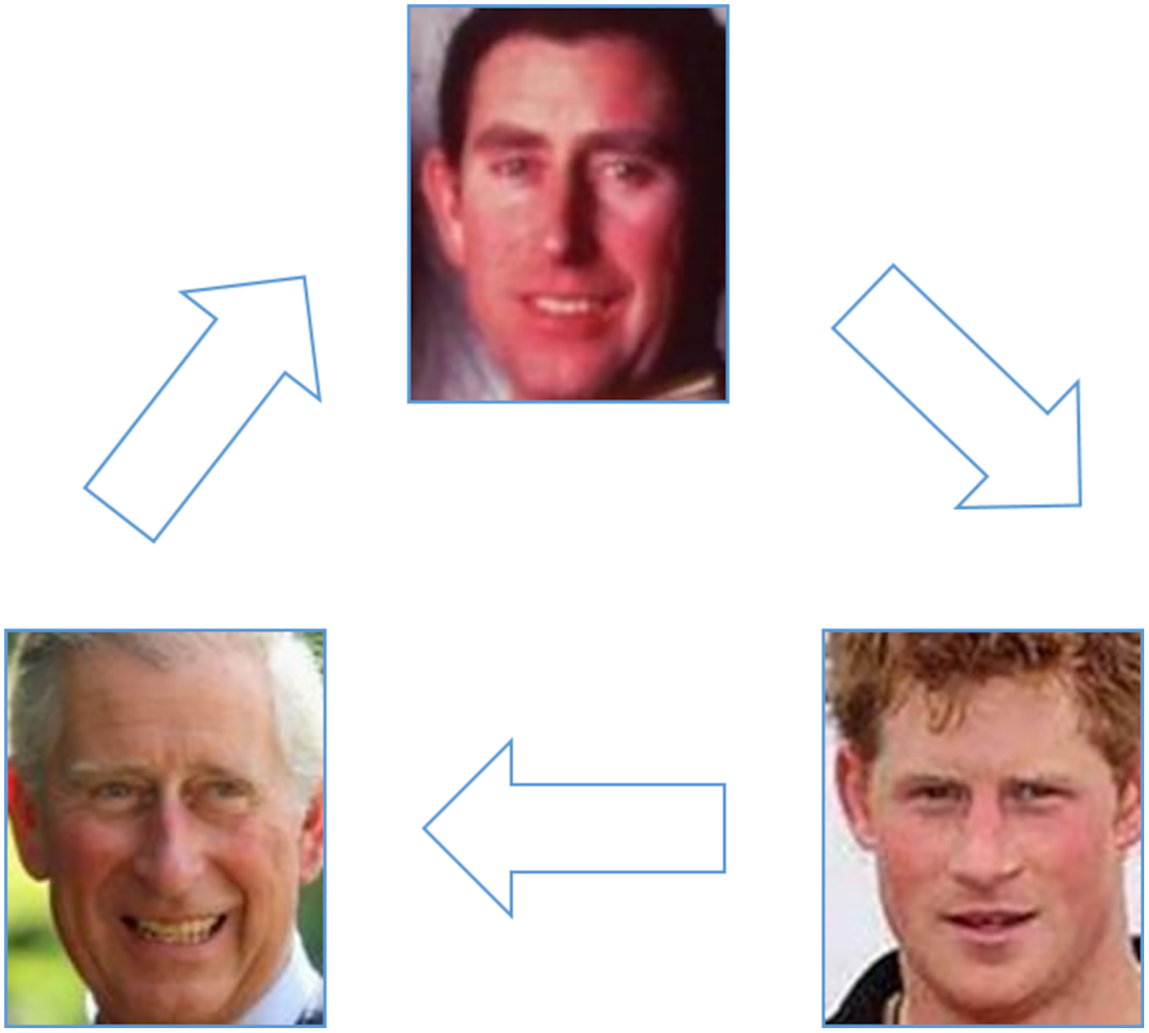
Kinship verification using mediator image (Ruogu et al., 2013).

Firstly, as mentioned before, kinship verification in age and gender variation is still a problem. For age variation, the previous studies (Shao, Xia & Fu, 2011; Xia, Shao & Fu, 2011; Xia et al., 2012) tried to minimize the broad gap between the children and their old parents by using the image of the old parents when they were young (*i.e*., in age similar to the current age of their children) as in UB KinFace dataset. In order to verify kinship between father and his son, Fig. 14 (the images are provided from Family 101 dataset (Fang et al., 2013)) shows an example of kinship verification of a father and his son using a mediator image of the father at his son’s age (on the top of the triangle). Because the mediator images are missing and existing dataset is not big enough., this solution is not so straightforward.

For gender variation, although most techniques and human trials have confirmed that gender plays a significant role in kinship verification, there is almost no special algorithm that targets addressing this issue. Although the solution to these issues is based on the development of computer vision and relevant fields, some encouraging efforts can be used for verifying kinship to design novel effective technologies for reducing defects caused by age and gender.

Second, kinship verification accuracy and efficiency were inconsistent in the established models. A cascade classifier construction and learning a model online are currently two main techniques of conflict mitigation. However, the first technique can’t guarantee the global optimization output, while the pattern presentation order easily influences the second one. Thus, developing technologies that include accuracy and efficiency requirements has a significant meaning to the advance of kinship verification. More effective models are expected to be seen in the next few years, such as Generative Adversarial Networks (GANS), adversarial machine learning, federated machine learning, and reinforcement learning.

GANs are a means to learn deep representations without having to use a lot of annotated data. These networks learn by deriving back propagation signals through a competitive process that involves two networks. The two networks are the generator $G$ and the Discriminator $D$. The $G$ is a network that is used to generate the images using random noise. The $D$ is considered as a discriminant network to determine whether a given image belongs to a real distribution or not. GANs can learn representations that can be employed in a variety of applications (Alqahtani, Kavakli-Thorne & Kumar, 2019).

Reinforcement Learning (RL) is a machine learning technique that enables an agent to learn through trial and error in an interactive environment while receiving feedback from its own actions and experiences. Rewarding desired behaviours and/or punishing undesirable ones are important to RL. A reinforcement learning agent can perceive and interpret its surroundings, execute actions, and learn through trial and error in general.

## Conclusions

The kin relation is one of the most common relations. Thus, kinship verification is considered a promising research area that requires more investigation. Despite having many common similarities and facing similar challenges, such as illumination and pose, kinship and face verification problems are addressed differently. It is due to the unique limitations that face kinship verification (*e.g*., gender) and that consequently cannot be solved using the current face verification techniques. Thus, developing simple and effective models to make accurate verification results gained an academic and practical interest. Over the last decade, many kinship researchers have conducted intensive research on this area of study from different angles to achieve high verification progress and enhancement. They focused on different ways of texture features extraction, combination of them and selection. This paper aims to present a comprehensive survey of current researches in kinship verification using facial image analysis. The paper illustrated KVR system limitations. Then, to get more information about kinship verification, the current state-of-the-art techniques have been reviewed. The reviewed techniques started from handcrafted, passing through shallow metric learning and ending up with the deep learning feature-based techniques in order to present the most efficient and flexible models. Furthermore, the performance of different models and techniques have been reviewed in different datasets. Finally, despite the scientific efforts that aim to address this hot research topic, many future research areas and techniques require investigation, such as using GANs, reinforcement learning variation, federated machine learning and adversarial machine learning to enhance kinship verification performance.

## Additional Information and Declarations

### Data Availability

The following information was supplied regarding data availability: The article used no data as it is a literature review.
